# Biomarkers and clinical scores to identify patient populations at risk of delayed antibiotic administration or intensive care admission

**DOI:** 10.1186/s13054-019-2613-4

**Published:** 2019-10-29

**Authors:** Juan Gonzalez del Castillo, Darius Cameron Wilson, Carlota Clemente-Callejo, Francisco Román, Ignasi Bardés-Robles, Inmaculada Jiménez, Eva Orviz, Macarena Dastis-Arias, Begoña Espinosa, Fernando Tornero-Romero, Jordi Giol-Amich, Veronica González, Ferran Llopis-Roca, on behalf of the INFURG-SEMES investigators, Joan Ramon Pérez-Mas, Elena Fuentes-González, Concepción Martínez-Muñoz, Elena Martínez-Beloqui, Francisco Javier Martín-Sánchez, Paula Mostaza Gallar, Luis Picazo García, Alejandro Malo de Molina Herrera

**Affiliations:** 1 Emergency Department, Hospital Clínico San Carlos, Madrid, Spain; 2 San Carlos Clinical Research Institute Hospital San Carlos (IdISSC), Madrid, Spain; 3 Shock, Organ Dysfunction and Resuscitation Research Group, Vall d’Hebron Institute of Research, Barcelona, Spain; 4 Emergency Department, Short Stay Unit and Home Hospitalization Unit, Hospital General de Alicante, Alicante, Spain; 5 Emergency Department, Hospital Universitari de Bellvitge, Barcelona, Spain; 6 Internal Medicine Department, Hospital Clínico San Carlos, Madrid, Spain; 7 Clinical Laboratory Department, Hospital Universitari de Bellvitge, Barcelona, Spain

**Keywords:** MR-proADM, Infection, Sepsis, NEWS, qSOFA, Disease progression, Emergency department, Intensive care unit

## Abstract

**Background:**

The performance of blood biomarkers (mid-regional proadrenomedullin (MR-proADM), procalcitonin (PCT), C-reactive protein (CRP), and lactate) and clinical scores (Sequential Organ Failure Assessment (SOFA), National Early Warning Score (NEWS), and quick SOFA) was compared to identify patient populations at risk of delayed treatment initiation and disease progression after presenting to the emergency department (ED) with a suspected infection.

**Methods:**

A prospective observational study across three EDs. Biomarker and clinical score values were calculated upon presentation and 72 h, and logistic and Cox regression used to assess the strength of association. Primary outcomes comprised of 28-day mortality prediction and delayed antibiotic administration or intensive care (ICU) admission, whilst secondary outcomes identified subsequent disease progression.

**Results:**

Six hundred eighty-four patients were enrolled with hospitalisation, ICU admission, and infection-related 28-day mortality rates of 72.8%, 3.4%, and 4.4%, respectively. MR-proADM and NEWS had the strongest association with hospitalisation and the requirement for antibiotic administration, whereas MR-proADM alone had the strongest association with ICU admission (OR [95% CI]: 5.8 [3.1 - 10.8]) and mortality (HR [95% CI]: 3.8 [2.2 - 6.5]). Patient subgroups with high MR-proADM concentrations (≥ 1.77 nmol/L) and low NEWS (< 5 points) values had significantly higher rates of ICU admission (8.1% vs 1.6%; *p* < 0.001), hospital readmission (18.9% vs. 5.9%; *p* < 0.001), infection-related mortality (13.5% vs. 0.2%; *p* < 0.001), and disease progression (29.7% vs. 4.9%; *p* < 0.001) than corresponding patients with low MR-proADM concentrations. ICU admission was delayed by 1.5 [0.25 – 5.0] days in patients with high MR-proADM and low NEWS values compared to corresponding patients with high NEWS values, despite similar 28-day mortality rates (13.5% vs. 16.5%). Antibiotics were withheld in 17.4% of patients with high MR-proADM and low NEWS values, with higher subsequent rates of ICU admission (27.3% vs. 4.8%) and infection-related hospital readmission (54.5% vs. 14.3%) compared to those administered antibiotics during ED treatment.

**Conclusions:**

Patients with low severity signs of infection but high MR-proADM concentrations had an increased likelihood of subsequent disease progression, delayed antibiotic administration or ICU admission. Appropriate triage decisions and the rapid use of antibiotics in patients with high MR-proADM concentrations may constitute initial steps in escalating or intensifying early treatment strategies.

## Background

Delayed treatment in patients presenting to the emergency department (ED) with a suspected infection may result in a prolonged hospitalisation, an increased morbidity, and a greater rate of infection-related mortality [1–3]. An accurate assessment of the severity of the host response and the potential for further disease progression and organ dysfunction is therefore crucial in order to administer a rapid and targeted therapeutic response.

The lack of validated tools to help guide therapeutic decision-making in patients presenting with low severity National Early Warning Score (NEWS) or quick Sequential Organ Failure Assessment (qSOFA) values, but with a high subsequent likelihood of further disease progression, is therefore of significant concern [4, 5]. Antibiotics are often administered before any final clinical diagnosis can be made [6], resulting in an increased likelihood of inappropriate therapy, growing levels of antibiotic resistance, and detrimental effects on the microbiota. Conversely, delayed treatment in high severity patients may lead to increased morbidity and mortality rates [7]. In addition, an early and inappropriate discharge from the ED may also result in higher mortality rates in patients later rehospitalised and directly admitted onto an intensive care unit (ICU), with similar findings also reported after an inappropriate initial admission onto a medical ward [8]. Thus, difficulties in identifying infection-related disease severity and the early pathophysiological changes involved in a deteriorating host response may contribute to poor overall decision-making and higher subsequent rates of hospital readmission [8–10].

Despite the presence of a number of independent risk factors [11–13], few studies have identified patient subgroups at risk of delayed antibiotic therapy or ICU triage, and the subsequent likelihood of further disease progression. It is therefore unsurprising that no validated test has been incorporated into routine clinical use. A recent investigation, however, found that the blood biomarker, mid-regional proadrenomedullin (MR-proADM), could accurately identify patients with non-severe clinical signs of infection but a high likelihood of further disease progression [4]. Indeed, recent studies have shown MR-proADM to improve National Early Warning Score (NEWS) performance in an undifferentiated ED population with mild clinical symptoms [14], identify disease progression in sepsis patients with decreasing procalcitonin (PCT) concentrations [15], and accurately identify non-surviving patients with low levels of organ dysfunction who later developed multiple organ failure [16].

This study therefore aimed to assess the potential use of MR-proADM in identifying high severity patient subgroups at risk of a delayed or insufficient initial treatment, identified by a decision to (i) withhold or delay antibiotic administration, or (ii) delay ICU admission. Biomarker kinetics between ED presentation and 72 h within patient subgroups were further investigated to identify subsequent cases of disease progression and mortality.

## Methods

### Study design and ethical approval

This prospective study consecutively enrolled patients presenting with a suspected infection to the EDs of three large tertiary level university hospitals (> 800 beds), comprising of the Hospital Clínico Universitario San Carlos (Madrid), the Hospital Universitari de Bellvitge (Barcelona), and the Hospital General Universitario de Alicante (Alicante). All patients were enrolled in accordance with the Helsinki Declaration, and ethical approval granted from the relevant governance bodies. The study was registered on ClinicalTrials.gov with the identifier NCT03992794.

### Inclusion and exclusion criteria

Inclusion criteria comprised of patients ≥ 18 years of age presenting with a clinical suspicion of infection as judged by the treating physician based on usual clinical practice, and could be made according to vital signs, main presenting symptoms, the request for a blood culture, or overall laboratory findings during standard ED assessment. Local study coordinators were responsible for collecting and recording all clinical data on a standardised case report form for each patient throughout the investigation. Exclusion criteria comprised of patients < 18 years of age, pregnancy, a refusal to participate, and no obvious clinical signs or symptoms of infection.

### Data collection and biomarker measurements

Patient demographics, comorbidities, initial diagnoses, and results from routine laboratory and microbiology tests were either recorded upon study enrolment or retrospectively added. CRP and lactate measurements were measured as part of the standard routine assessment, with a second blood draw taken from each patient during the initial clinical assessment to measure PCT and MR-proADM concentrations using a non-commercially available point-of-care duplex biomarker device (Samsung LABGEO IB10, Nexus, USA). The location of each patient after 72 h was further identified, with both clinical data and an additional blood sample for PCT and MR-proADM measurement taken in patients still hospitalised at this time point. Samples were measured within 15 min of being drawn by the study coordinator at each site; thus, neither PCT nor MR-proADM results were made available to the treating physician throughout patient enrolment or hospitalisation. Survival and ICU admission time was censored at 28 days following ED presentation, and patients discharged prior to this time point were subsequently contacted by phone to ascertain survival status.

### Study endpoints

Study endpoints were defined as follows: *antibiotic administration *— administration of intravenous, oral, or intramuscular antibiotics during ED treatment; *length of time to antibiotic administration* — length of time from arrival in the ED to the first administration of antibiotics; *delayed antibiotic administration* — initiation of antibiotic therapy ≥ 180 min following arrival in the ED [17, 18]; *hospitalisation *— hospital admission with a subsequent stay of > 24 h; *intensive care unit (ICU) admission *— all-cause ICU admission within 28 days of study enrolment which could be further categorised into three categories: immediate (0 days - same day as ED presentation), delayed (between 1 and 7 days following ED presentation), and late (> 7 days following presentation); *hospital readmission *— readmission due to an infection-related symptom or cause within 28 days following ED or hospital discharge; *28-day mortality* — mortality within 28 days due to an infection-related cause; and *disease progression *— composite endpoint consisting of infection-related 28-day mortality, ICU admission, and a ≥ 2 point increase in NEWS or SOFA score between presentation and 72 h.

### Primary and secondary outcomes

Primary study outcomes comprised of 28-day mortality prediction and the identification of patient populations enriched for a delayed (i) antibiotic administration, or (ii) ICU admission. Secondary outcomes comprised of patient populations enriched according to (iii) subsequent disease progression.

### Statistical analysis

Symmetrically distributed data were reported using mean and standard deviation values, whilst skewed data reported using median, first quarter, and third quarter values. Demographic and clinical data were assessed using the chi-square (*χ*^2^) for categorical variables, and either Student’s *t* test or the Mann-Whitney *U* test for symmetrical or skewed continuous variables, respectively. Receiver operating characteristic (ROC) and areas under the curve (AUC) determined the predictive value of each parameter for 28-day mortality, antibiotic administration, hospitalisation, and ICU admission decisions, with 95% confidence intervals (95% CI) used to determine significance. Optimised cut-off values for sensitivity and specificity were determined using Youden’s criterion, and patient subgroups subsequently identified according to optimised cut-off values for the prediction of 28-day mortality, similar to methods outlined by Saeed et al. [4]. Kaplan-Meier curves categorised patients on ED presentation according to either optimised or pre-established 28-day mortality cut-offs for all biomarkers and scores, with the most accurate parameter used to further stratify subgroups. Treatment and outcome characteristics of each resulting subgroup, comprising of antibiotic, hospitalisation, disease progression, intensive care, and mortality-related variables, were compared using the log-rank test for mortality; the chi-square (*χ*^2^) test for disease progression, hospitalisation, ICU admission, and antibiotic administration; and the Mann-Whitney U test for the overall length of hospitalisation. Univariate and multivariate Cox regression analyses assessed the association of each parameter with time to mortality, whilst corresponding logistic regression assessed the association with antibiotic administration, hospitalisation, and ICU admission decisions. Potential confounding variables were selected based on a univariate survival analysis for infection-related 28-day mortality and subsequently included in all further multivariate analyses as adjusting variables. Results were presented as either the hazard (HR) or the odds (OR) ratio per 1 interquartile-range increase for Cox and logistic regression analyses, respectively. A *p* value < 0.05 was considered statistically significant, and all data analysed using the statistics software R (version 3.1.2). Due to the exploratory nature of the primary and secondary endpoints, no a priori sample size calculation could be performed.

## Results

### Patient characteristics

**Fig. 1:**
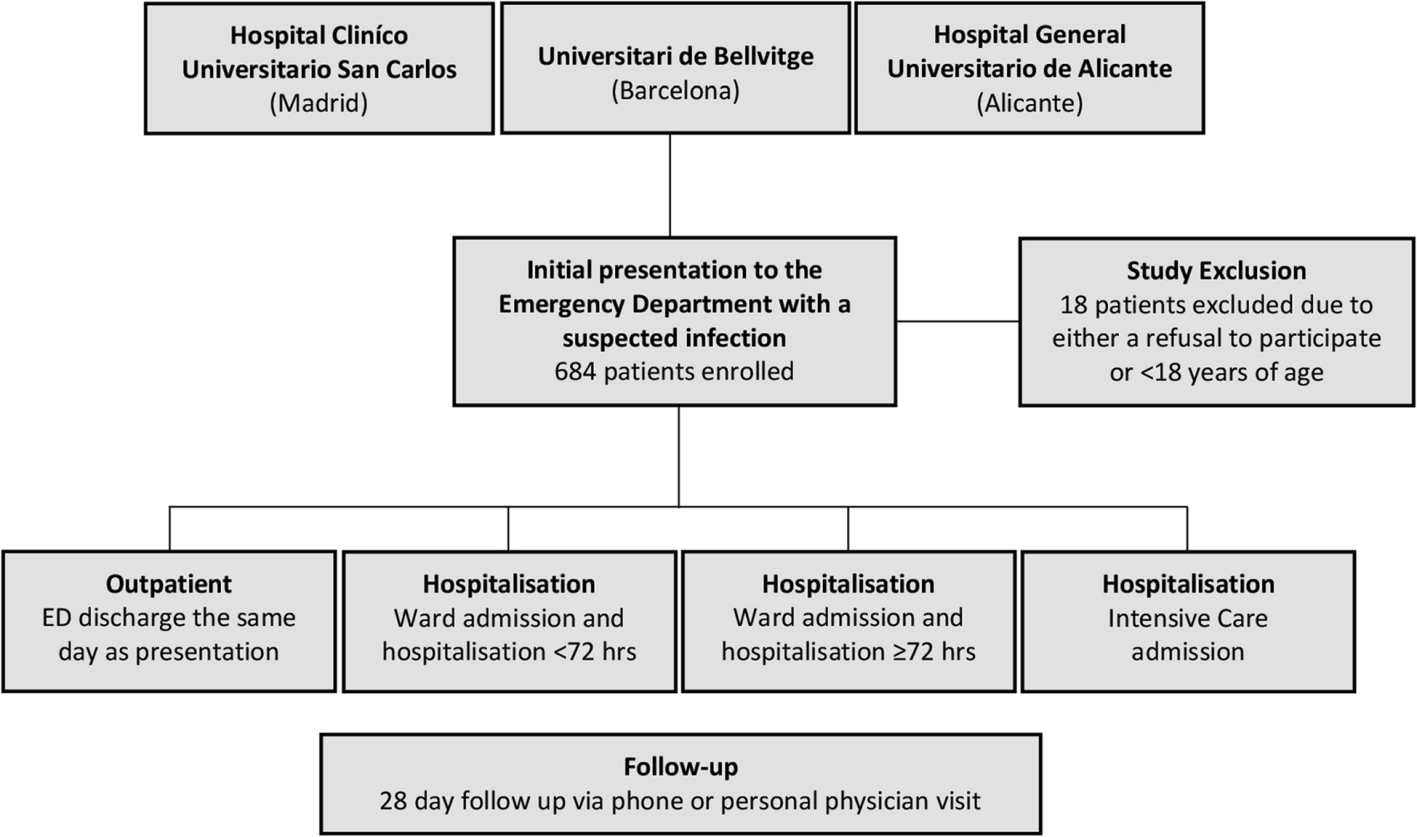
Patient enrolment and follow-up process

A total of 684 patients with suspected infection were consecutively enrolled between May and July 2018 (Fig. 1), with lower respiratory tract and urogenital infections the most common origins of infection (Table 1; Additional file 1: Table S1). Only 55.6% (*N* = 380) of patients remained hospitalised after 72 h following ED presentation (Additional file 1: Table S2).

**Table 1:** Patient characteristics in the total population and with regard to infection-related 28-day mortality

Patient characteristics	Total cohort *(N* = 684)	Survivors (*N* = 654)	Non-survivors (*N* = 30)	*p* value
Demographics				
Age (years)	65.1 (19.6)	64.4 (19.8)	78.9 (9.0)	< 0.001
Male sex (*N*, %)	366 (53.5%)	352 (53.8%)	14 (46.7%)	0.442
Disposition				
Hospital admission (*N*, %)	498 (72.8%)	469 (71.7%)	29 (96.7%)	0.003
Hospital length of stay (days)	3 [0 – 7.25]	3 [0 – 7]	12 [3 – 20.5]	< 0.001
ICU admission (*N*, %)	23 (3.4%)	19 (2.9%)	4 (13.3%)	0.002
Comorbidities				
Cardiovascular disease (*N*, %)	255 (37.3%)	239 (36.5%)	16 (53.3%)	0.063
Diabetes (*N*, %)	152 (22.2%)	140 (21.4%)	12 (40.0%)	0.017
Immunodeficiency (*N*, %)	105 (15.4%)	97 (14.8%)	8 (26.7%)	0.079
Liver disease (*N*, %)	54 (7.9%)	48 (7.3%)	6 (20.0%)	0.012
Malignancy (*N*, %)	183 (26.8%)	169 (25.8%)	14 (46.7%)	0.012
Neurological disorders (*N*, %)	132 (19.3%)	125 (19.1%)	7 (23.3%)	0.567
Respiratory disease (*N*, %)	180 (26.3%)	173 (26.5%)	7 (23.3%)	0.704
Renal disease (*N*, %)	128 (18.7%)	117 (17.9%)	11 (36.7%)	0.010
Infectious source				
Bone and joint (*N*, %)	4 (0.6%)	3 (0.5%)	1 (3.3%)	0.044
Cardiac (*N*, %)	2 (0.3%)	1 (0.2%)	1 (3.3%)	0.002
Intra-abdominal (*N*, %)	93 (13.6%)	86 (13.1%)	7 (23.3%)	0.112
Respiratory — lower (*N*, %)	220 (32.2%)	206 (31.5%)	14 (46.7%)	0.082
Respiratory — upper (*N*, %)	18 (2.6%)	18 (2.8%)	0 (0.0%)	0.357
Skin and soft tissue (*N*, %)	36 (5.3%)	35 (5.4%)	1 (3.3%)	0.628
Surgical related (*N*, %)	16 (2.3%)	16 (2.4%)	0 (0.0%)	0.386
Unknown origin (*N*, %)	81 (11.8%)	80 (12.2%)	1 (3.3%)	0.140
Urogenital (*N*, %)	214 (31.3%)	209 (32.0%)	5 (16.7%)	0.077
Blood cultures				
Blood cultures taken (*N*, %)	407 (59.5%)	390 (59.6%)	17 (56.7%)	0.746
Positive blood cultures (*N*, %)	58 (14.3%)	55 (8.4%)	3 (10.0%)	0.756
Gram-positive bacteria (*N*, %)	15 (2.2%)	14 (2.1%)	1 (3.3%)	0.326
Gram-negative bacteria (*N*, %)	35 (5.1%)	33 (5.0%)	2 (6.7%)	0.762
Gram-positive and gram-negative bacteria (*N*, %)	8 (1.2%)	7 (1.1%)	1 (3.3%)	0.737
Biomarkers and severity scores				
MR-proADM (nmol/L)	1.09 [0.70 – 1.71]	1.05 [0.68 – 1.64]	2.32 [1.89 – 2.96]	< 0.001
PCT (ng/mL)	0.21 [0.10 – 0.98]	0.2 [0.10 – 0.90]	0.59 [0.21 – 3.78]	< 0.001
Lactate (mmol/L)	1.5 [1.1 – 2.1]	1.5 [1.1 – 2.1]	2.1 [1.58 – 2.83]	< 0.01
CRP (mg/L)	10.52 [3.17 – 26.3]	10.52 [3.12 – 25.63]	9.99 [6.36 – 18.35]	0.925
SOFA (points)	1 [0 – 3]	1 [0 – 3]	4 [3 – 5]	< 0.001
qSOFA (points)	0 [0 – 1]	0 [0 – 1]	1 [1 – 1]	< 0.05
SIRS (points)	1 [1 – 2]	1 [1 – 2]	2 [1 – 2]	< 0.001
NEWS (points)	2 [1 – 5]	2 [1 – 5]	6 [3.25 – 7.75]	< 0.001
CRB-65 (points)	1 [0 – 2]	1 [0 – 1]	2 [1 – 2]	< 0.001

Values expressed in percentages (%) indicate the proportion of patients within each cohort for each variable. Data are presented as mean (standard deviation, SD) or median [first quartile (Q1)–third quartile (Q3)] where specified. The chi-square (*χ*^2^) test was used to determine significance between the cohorts for categorical variables, Student’s *t* test for the variable of age, and Mann-Whitney *U* test for hospitalisation duration, biomarker, and clinical score variables. *CRB-65* severity score for community-acquired pneumonia, *CRP* C-reactive protein, *ICU* intensive care unit, *MR-proADM* mid-regional proadrenomedullin, *N* number, *NEWS* National Early Warning Score, *PCT* procalcitonin, *qSOFA* quick Sequential Organ Failure Assessment, *SIRS* systemic inflammatory response syndrome, *SOFA* Sequential Organ Failure Assessment

### Treatment decisions within the total population

**Fig. 2:**
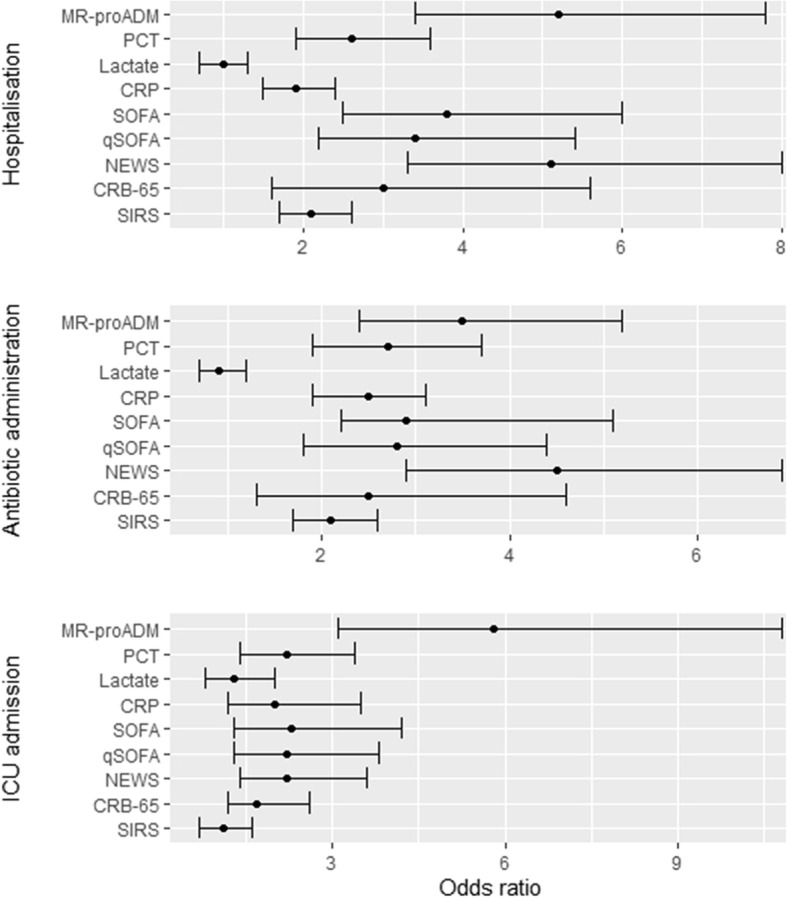
Multivariate logistic regression for all biomarkers and clinical scores for antibiotic administration, hospitalisation, and intensive care (ICU) admission endpoints. CRB-65, severity score for community-acquired pneumonia; CRP, C-reactive protein; MR-proADM, mid-regional proadrenomedullin; NEWS, National Early Warning Score; PCT, procalcitonin; SIRS, systemic inflammatory response syndrome; qSOFA, quick Sequential Organ Failure Assessment; SOFA, Sequential Organ Failure Assessment

Antibiotics were administered to 73.8% (*N* = 505) of patients during treatment within the ED, with 15.2% (*N* = 104) already undergoing therapy prior to presentation. The median time from presentation to administration was 186 (120 – 330) min, with the decision to hospitalise concurrently made in 72.8% (*N* = 498) of patients (Table 1). ICU admission was required in 3.4% (*N* = 23) of patients, with 47.8% (*N* = 11) of admissions occurring on the same day as ED presentation. The overall median time to admission was 1 (0 – 11.5) day. Univariate logistic regression found that NEWS and MR-proADM had the strongest association with antibiotic administration (NEWS vs. MR-proADM OR [95% CI] 5.5 [3.6 – 8.2] vs. 4.9 [3.5 – 6.8]; Additional file 1: Table S3) and hospitalisation (NEWS vs. MR-proADM OR [95% CI] 6.0 [4.0 – 9.1] vs. 6.6 [4.6 – 9.4]; Additional file 1: Table S5) decisions, whereas MR-proADM alone had the highest association with the requirement for ICU admission (OR [95% CI] 4.1 [2.3 – 7.1]; Additional file 1: Table S7). Multivariate analysis was adjusted by age, diabetes, malignancy, and liver and renal disease variables, with the association for NEWS and MR-proADM maintained for each endpoint (Fig. 2; Additional file 1: Tables S3, S5, and S7). Addition of MR-proADM to each of the other biomarkers and scores significantly improved association for each endpoint (Additional file 1: Tables S4, S6, and S8). Corresponding AUROC analysis found similar results, with MR-proADM having the greatest accuracy and the highest diagnostic odds ratio for each endpoint (Additional file 1: Figures S1–S3).

### Subgroup enrichment for infection-related mortality upon ED presentation and 72 h

**Fig. 3:**
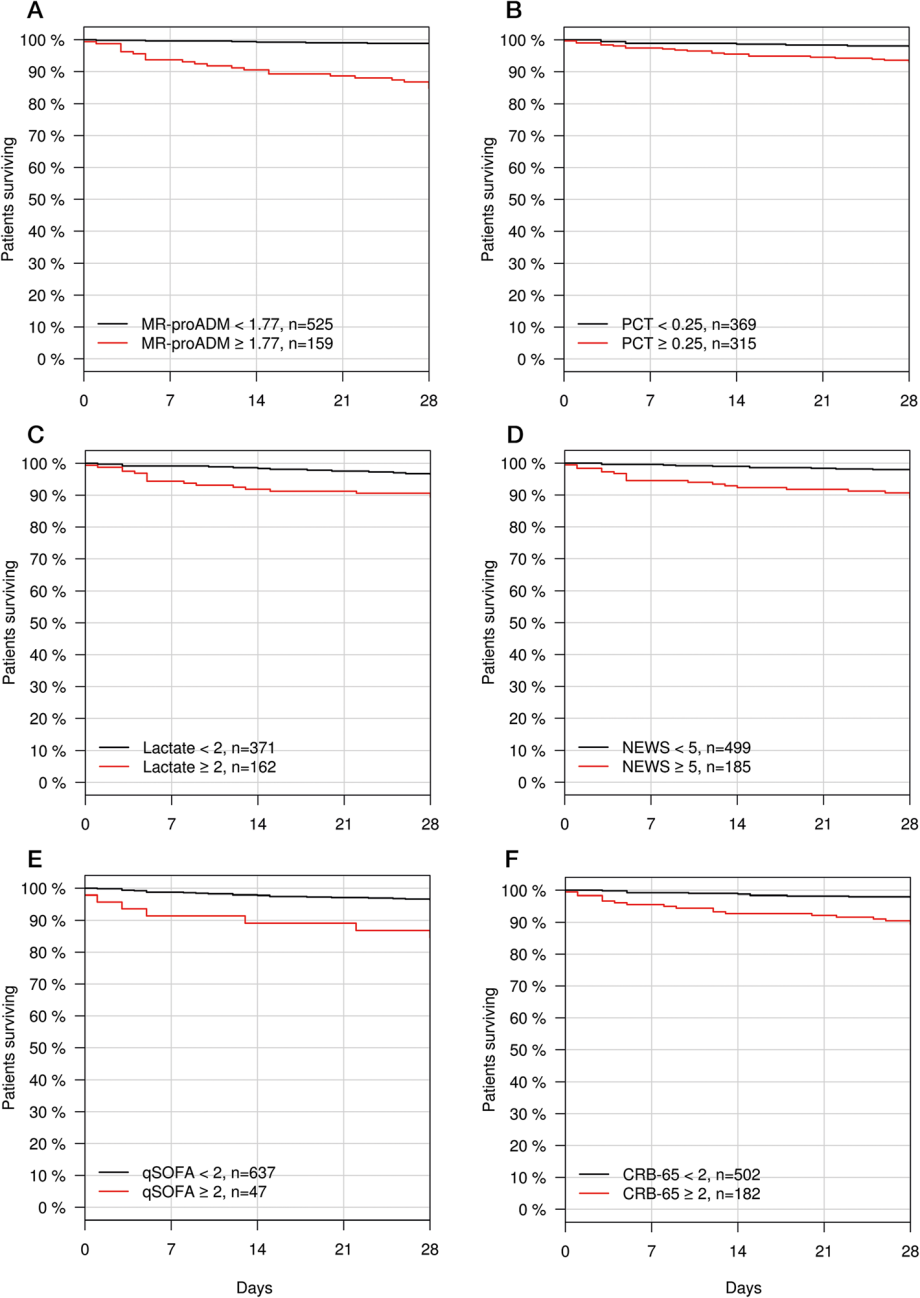
Kaplan-Meier analysis to identify disease severity subgroups using biomarkers and clinical scores according to MR-proADM (**a**), PCT (**b**), lactate (**c**), NEWS (**d**), qSOFA (**e**), and CRB-65 (**f**) cut-offs. CRB-65, severity score for community-acquired pneumonia; MR-proADM, mid-regional proadrenomedullin; NEWS, National Early Warning Score; qSOFA, quick Sequential Organ Failure Assessment

The infection-related 28-day mortality rate upon presentation and 72 h was 4.4% (*N* = 30; Table 1) and 6.8% (*N* = 26; Additional file 1: Table S2), respectively. Non-survivors were significantly older and had a median time to mortality of 11 (4.25 – 19.50) days, with the most common cause of death being either single or multiple organ failure (*N* = 22; 73.3%). Non-infected related mortalities (*N* = 4) comprised of cerebral haemorrhage and issues relating to a prior trauma. All biomarkers and clinical scores were significantly increased in the non-surviving population, with the exception of CRP (Table 1). Univariate (Additional file 1: Tables S9 and S11) and multivariate (Table 2) Cox regression found that MR-proADM had the greatest association with mortality upon presentation and 72 h, although the strength of association decreased between both time points. Addition of MR-proADM to each of the other biomarkers and scores significantly improved association at both time points (Additional file 1: S10 and S12). Corresponding AUROC analysis found similar results (Additional file 1: Figure S4), with an MR-proADM cut-off of 1.77 nmol/L at presentation resulting in the highest prognostic odds ratio. Kaplan-Meier curve analysis found that MR-proADM could most accurately identify low and high disease severity populations compared to other biomarkers or scores upon presentation (Fig. 3; Additional file 1: Tables S13 and S14) and after 72 h (Additional file 1: Tables S15 and 16).

**Table 2:** Multivariate Cox regression analysis for the prediction of 28-day mortality upon ED presentation and after 72 h of hospitalisation

Biomarker or clinical score	Patients (*N*)	Number of events (*N*)	LR *χ*^2^	*p* value	HR [95% CI]
28-day mortality prediction upon ED presentation					
MR-proADM	684	30	53.1	< 0.001	3.8 [2.2 – 6.5]
PCT	684	30	38.2	< 0.001	1.7 [1.1 – 2.5]
Lactate	533	30	34.8	< 0.001	1.5 [1.1 – 2.1]
CRP	646	28	29.0	< 0.001	0.9 [0.6 – 1.4]
SOFA	684	30	53.7	< 0.001	3.2 [2.1 – 5.0]
qSOFA	684	30	46.5	< 0.001	2.3 [1.6 – 3.5]
NEWS	684	30	42.2	< 0.001	2.0 [1.3 – 2.9]
CRB-65	684	30	39.8	< 0.001	1.5 [1.1 – 2.0]
SIRS	684	30	35.4	< 0.001	1.4 [1.0 – 1.9]
28-day mortality prediction after 72 h of hospitalisation					
MR-proADM	375	25	36.6	< 0.001	3.0 [1.8 – 4.9]
PCT	370	25	26.2	< 0.001	1.6 [1.1 – 2.4]
Lactate	266	24	20.1	< 0.01	1.0 [0.7 – 1.5]
CRP	281	20	17.6	< 0.001	0.8 [0.4 – 1.3]
SOFA	375	25	33.7	< 0.001	2.0 [1.4 – 2.7]
qSOFA	376	25	22.5	< 0.001	1.4 [0.8 – 2.3]
NEWS	376	25	26.5	< 0.001	1.6 [1.1 – 2.3]
CRB-65	376	25	22.3	< 0.001	1.3 [0.8 – 2.2]
SIRS	376	25	24.9	< 0.001	1.5 [1.0 – 2.3]

Multivariate analysis was adjusted by age, diabetes, malignancy, and liver and renal disease variables. *CI* confidence interval, *CRB-65* severity score for community-acquired pneumonia, *CRP* C-reactive protein, *DF* degrees of freedom, *LR* likelihood ratio, *MR-proADM* mid-regional proadrenomedullin, *N* number, *NEWS* National Early Warning Score, *HR* hazard ratio, *PCT* procalcitonin, *qSOFA* quick Sequential Organ Failure Assessment, *SIRS* systemic inflammatory response syndrome, *SOFA* Sequential Organ Failure Assessment

**Fig. 4:**
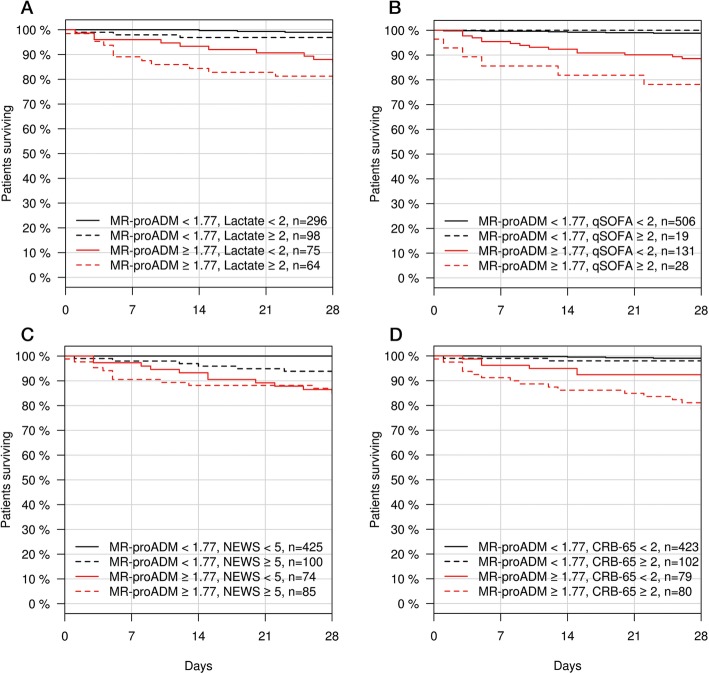
Kaplan-Meier analysis to identify patient populations enriched for disease progression events. Patients were stratified according to a combination of MR-proADM and lactate (**a**), qSOFA (**b**), NEWS (**c**), and CRB-65 (**d**). CRB-65, severity score for community-acquired pneumonia; MR-proADM, mid-regional proadrenomedullin; NEWS, National Early Warning Score; qSOFA, quick Sequential Organ Failure Assessment

In patients with low NEWS values (< 5 points, *N* = 499), MR-proADM was found to have the greatest performance of all biomarkers in predicting 28-day mortality (*N* = 10; 2.0%; AUROC [95% CI] 0.95 [0.92 – 0.98]), followed by PCT and lactate (AUROC [95% CI] 0.78 [0.70 – 0.86] and 0.72 [0.56 – 0.87], respectively). The presence of low NEWS and high MR-proADM (≥ 1.77 nmol/L) values on ED presentation resulted in a patient subgroup (*N* = 75; 11.3%) enriched for cases of infection-related mortality (Fig. 4C), with similar subgroups (e.g. low lactate, qSOFA, and CRB-65) found for other biomarkers and clinical scores (Fig. 4).

### Subgroup enrichment for withheld or delayed antibiotic therapy

The proportion of patients receiving antibiotics < 180 mins after ED arrival was significantly (*p* < 0.01) lower in the high MR-proADM and low lactate (*N* = 32; 45.7%) as opposed to the high MR-proADM and high lactate (*N* = 38; 66.7%) subgroup, despite similar rates of infection-related readmission and mortality, ICU admission, and disease progression (Additional file 1: Table S17). Comparable results could also be found for MR-proADM and qSOFA subgroups (Additional file 1: Table S18), whereas no such trend could be found for MR-proADM and either NEWS (Table 3) or CRB-65 (Additional file 1: Table S19).

**Table 3:** Patient subgroups stratified by NEWS and MR-proADM

Patient subgroups	Patient populations stratified by NEWS and MR-proADM							
	MR-proADM (nmol/L)	NEWS (points)	MR-proADM (nmol/L)	NEWS (points)	MR-proADM (nmol/L)	NEWS (points)	MR-proADM (nmol/L)	NEWS (points)
	< 1.77	< 5	≥ 1.77	< 5	< 1.77	≥ 5	≥ 1.77	≥ 5
Population, *N* (%)	425 (62.1%)		74 (10.8%)		100 (14.6%)		85 (12.8%)	
Antibiotic administration, *N* (%)	266 (62.6%)		63 (85.1%)		94 (94.0%)		82 (96.5%)	
Time to antibiotic administration (min) (median [Q1–Q4])	180 [90 – 342]		205.5 [111 – 300]		210 [142 – 315]		180 [100 – 300]	
Antibiotic administration < 180 min, *N* (%)	108 (40.6%)		26 (41.3%)		30 (31.9%)		33 (40.2%)	
i.v. to oral antibiotic change (days) (median [Q1–Q4])	1.5 [1 – 4]		4.0 [0 – 6.75]		4 [2 – 6]		3.5 [0 – 4.5]	
Hospitalisation, *N* (%)	260 (61.2%)		63 (85.1%)		93 (93.0%)		82 (96.5%)	
Length of hospitalisation (days) (median [Q1–Q4])	1 [0 – 5]		6 [3 – 11]		7 [4 – 10]		7 [4 – 13]	
Infection-related readmission, *N* (%)	25 (5.9%)		14 (18.9%)		3 (3.0%)		9 (10.6%)	
ICU admission, *N* (%)	7 (1.6%)		6 (8.1%)		2 (2.0%)		8 (9.4%)	
Time to ICU admission (days) (median [Q1–Q4])	13 [2 – 20]		1.5 [1 – 5]		9.5 [4.75 – 14.25]		0 [0 – 0]	
Number of immediate ICU admissions, *N* (%)	0 (0.0%)		0 (0.0%)		0 (0.0%)		7 (30.4%)	
Number of delayed ICU admissions, *N* (%)	2 (8.7%)		5 (21.7%)		1 (4.3%)		0 (0.0%)	
Number of late ICU admissions, *N* (%)	5 (21.7%)		1 (4.3%)		1 (4.3%)		1 (4.3%)	
Infection-related 28-day mortality, *N* (%)	0 (0.0%)		10 (13.5%)		6 (6.0%)		14 (16.5%)	
Hospital mortality, *N* (%)	1 (0.2%)		10 (13.5%)		9 (9.0%)		15 (17.6%)	
Disease progression, *N* (%)	21 (4.9%)		22 (29.7%)		12 (12.0%)		24 (28.2%)	

Number of immediate, delayed, or late ICU admissions are expressed as a percentage of the total number of ICU admissions. *ICU* intensive care unit, *i.v.* intravenous, *MR-proADM* mid-regional proadrenomedullin, *N* number, *NEWS* National Early Warning Score

Nevertheless, patients with low NEWS and low MR-proADM values could be further categorised according to whether antibiotics were administered or withheld during ED treatment, with no significant differences found in subsequent ICU admission, hospital readmission, or infection-related mortality rates irrespective of antibiotic administration (Additional file 1: Table S20). Conversely, antibiotic administration in the low NEWS and high MR-proADM subgroup resulted in significantly lower ICU admission (*N* = 3, 4.8% vs. *N* = 3, 27.3%; *p* < 0.001) and hospital readmission rates (*N* = 9, 14.3% vs. *N* = 6, 54.5%; *p* < 0.001) compared to corresponding patients where therapy was withheld (Additional file 1: Table S20). Similar results were also observed for MR-proADM and qSOFA subgroups depending on antibiotic administration (Additional file 1: Table S21).

### Subgroup enrichment for delayed ICU admission

Patients with low NEWS and high MR-proADM values had a significantly higher risk of overall as well as delayed ICU admission compared to corresponding patients with low MR-proADM concentrations (*p* < 0.001), with an average time to admission of 1.5 [0.25 – 5] days (Table 3). This comprised of 2 patients initially deemed suitable for outpatient treatment, but later directly admitted onto the ICU after re-presenting to the ED, as well as 4 patients initially triaged onto a medical ward before subsequent ICU admission. Conversely, no significant differences in admission rate were found compared to the high NEWS and high MR-proADM subgroup (*N* = 8; 9.4%; Table 3), where all admissions were immediately transferred onto the ICU. Similar findings could also be found for MR-proADM and qSOFA subgroups.

### Subgroup enrichment for disease progression and biomarker kinetics to identify non-surviving patients

Low NEWS and high MR-proADM concentrations on ED presentation resulted in a patient subgroup enriched for cases of subsequent disease progression (Table 3), with similar results also found for combinations of high MR-proADM and low lactate, qSOFA, and CRB-65 (Additional file 1: Tables S17–S19). Interestingly, no significant kinetical differences were found in surviving or non-surviving patients with initially low NEWS values between presentation and 72 h, whilst MR-proADM significantly decreased in survivors (*p* < 0.001) and tended to increase in non-survivors (*p* = 0.085; Table 4). Such a kinetical profile was not observed for any other biomarker in patients with initially low NEWS values (Additional file 1: Table S22). Similar subgroup enrichment and comparably increased or continuously elevated kinetics between presentation and 72 h could also be found in high MR-proADM and low qSOFA (Additional file 1: Table S23), lactate, and CRB-65 subgroups.

**Table 4:** NEWS and MR-proADM values upon ED presentation and 72 h within patient subgroups

Patient subgroups	Patient populations stratified by NEWS and MR-proADM							
	MR-proADM (nmol/L)	NEWS (points)	MR-proADM (nmol/L)	NEWS (points)	MR-proADM (nmol/L)	NEWS (points)	MR-proADM (nmol/L)	NEWS (points)
	< 1.77	< 5	≥ 1.77	< 5	< 1.77	≥ 5	≥ 1.77	≥ 5
Population, *N* (%)	425 (62.1%)		74 (10.8%)		100 (14.6%)		85 (12.8%)	
Infection related 28-day mortality, *N* (%)	0 (0.0%)		10 (13.5%)		6 (6.0%)		14 (16.5%)	
NEWS: surviving patients								
ED admission (points)	1.72 (1.29)		2.18 (1.39)		7.18 (1.71)		7.97 (2.62)	
72 h (points)	1.54 (1.62)		2.08 (2.26)		4.57 (3.22)		3.61 (2.48)	
*p* value	0.182		0.784		< 0.001		< 0.001	
NEWS: non-surviving patients								
ED admission (points)	NA		2.60 (0.97)		6.20 (1.10)		8.36 (2.69)	
72 h (points)	NA		2.63 (2.07)		4.20 (2.03)		4.90 (3.31)	
*p* value	NA		0.875		< 0.05		< 0.05	
MR-proADM: surviving patients								
ED admission (nmol/L)	0.88 (0.36)		2.78 (1.16)		1.23 (0.34)		3.39 (1.47)	
72 h (nmol/L)	0.91 (0.58)		1.80 (1.11)		1.08 (0.50)		2.13 (1.36)	
*p* value	0.56		< 0.001		< 0.01		< 0.001	
MR-proADM: non-surviving patients								
ED admission (nmol/L)	NA		3.22 (1.37)		1.30 (0.29)		2.77 (1.38)	
72 h (nmol/L)	NA		3.95 (1.02)		1.41 (0.35)		3.69 (2.49)	
*p* value	NA		0.085		0.548		0.148	

All NEWS and MR-proADM values are expressed as mean (standard deviation). *MR-proADM* mid-regional proadrenomedullin, *N* number, *NEWS* National Early Warning Score

## Discussion

The results of this prospective multicentre study confirm those from previous investigations [4, 19], highlighting the ability of MR-proADM to identify patients with a high potential for subsequent disease progression [15, 16, 20]. Results also indicate that patients with low presenting symptoms and high MR-proADM concentrations had an increased risk of a less intensive treatment despite high subsequent mortality rates, characterised by a withheld or delayed antibiotic therapeutic response, a delayed admission onto the ICU, and a high readmission rate due to the reoccurrence of an infection-related complication.

Treatment during ED assessment is often initiated before any definitive diagnosis can be made in order to limit the potential for subsequent clinical deterioration [6]. This, however, may be complicated by the heterogeneous and multifaceted host response to infection [21], as well as difficulties in assessing the severity and potential for further disease progression. There is therefore a high likelihood of either an over- or an under-treatment of patients, both of which are associated with undesirable outcomes. Hence, the use of an easily measurable parameter to accurately assess infection severity and short-term disease progression is highly desirable in order to help guide optimal treatment decision-making. Based on recent evidence, the blood biomarker mid-regional proadrenomedullin (MR-proADM) may potentially fulfil this unmet clinical need, with elevated concentrations found due to increased capillary leak and deteriorating microcirculatory integrity [22–24]. Such pathophysiological changes, however, are not unique to patients with infection. Indeed, elevated MR-proADM concentrations have been observed across of range of non-infection-related conditions, such as acute and chronic heart failure [25, 26], non-specific complaints [27], and in the build-up to acute episodes of systemic capillary leak syndrome (Clarkson’s disease) [28]. Thus, any increase in MR-proADM concentration in patients with suspected infection cannot be specifically attributed to the presence of an infectious source, although it may provide an early and accurate prediction of developing organ dysfunction and subsequent mortality [29–34]. Thus, the fundamental challenge in incorporating such a parameter into routine clinical practice therefore relates to the extent to which real life decision-making can either be altered or optimised.

Three potential areas of further observational and interventional research using MR-proADM can therefore be proposed based on the results of this study, as well as current clinical requirements, namely as a potential aid to (i) guide appropriate and timely antibiotic administration, (ii) minimise the risk of inappropriate triage before admission onto an intensive care unit, and (iii) identify treatment failure and disease progression in patients with low severity clinical symptoms.

Firstly, the early administration of oral or intravenous antibiotics in patients presenting with a suspected infection plays a central role in most emergency medicine treatment strategies. However, challenges concerning their unnecessary administration are well documented, primarily due to increasing antibiotic resistance and rising healthcare costs. Conversely, the importance of ensuring therapy is rapidly administered to both high severity patients and those with a high potential for further disease progression cannot be overstated [6, 35–38]. As such, no standardised strategy exists to guide antibiotic administration in the ED. Interestingly, our results highlight a greater association with the requirement for antibiotic administration using NEWS and MR-proADM, as opposed to more commonly used parameters such as CRP and PCT, which confirm the results of a recent subset analysis [39] from a previous study. This may be in part explained by the rapid kinetical profile of MR-proADM, which is increased significantly earlier than PCT [40, 41] and many other cytokines [42] in response to microbial infection. Nevertheless, elevated MR-proADM concentrations can also be observed in many non-infectious conditions, albeit to a lesser extent than during a severe infectious episode, making its sole use in guiding antibiotic administration problematic. The combination of PCT and MR-proADM, therefore, may provide an attractive alternative [43–45].

Secondly, the rapid triage of high severity patients onto the ICU is mandatory in order to prevent further disease progression and maximise the chances of a successful treatment. Nevertheless, many patients are inappropriately triaged following ED presentation back into the community or onto a medical ward before any subsequent ICU admission, thus increasing the likelihood of a prolonged hospitalisation or ultimate mortality [8, 46, 47]. Our results suggest that the presence of low severity vital and physiological signs of infection may create a false impression concerning the requirement for immediate ICU admission, whilst elevated MR-proADM concentrations — indicative of the early stages of developing organ dysfunction — may provide significant additional information in order to optimise decision-making. Similar findings have also previously been reported to predict renal replacement therapy (RRT) requirement in patients where no RRT was previously initiated [20] and in patients who progressed towards multiple organ failure [15, 16].

Finally, the results of this study suggest that MR-proADM can accurately identify specific patient subgroups based on the likelihood of further clinical deterioration, thus helping to optimise subsequent treatment and triage decision-making. Results confirm those of a previous investigation [4], whilst further patient evaluation at 72 h found that MR-proADM was the only parameter to significantly decrease in surviving patients with low clinical scores, whilst tending to increase in non-survivors. Similar results have been found in previous investigations where continuously high or increasing MR-proADM concentrations in the ED [48] or ICU [15] were indicative of a subsequent detrimental outcome, or the requirement for an urgent clinical intervention.

We note several limitations and strengths of this study that deserve greater discussion. Firstly, a significant number of non-surviving patients showed no clinical deterioration at 72 h according to the calculation of clinical score values. Consecutive measurements should therefore be made across further time points to fully capture any subsequent deterioration. Nevertheless, such a finding is likely to be of relative clinical interest, since continuously elevated or increasing MR-proADM concentrations between both time points may potentially provide an earlier warning of treatment failure than conventional clinical scores. Secondly, numerous factors such as ED waiting times and physician availability may influence the timing of initial antibiotic administration [17], thus contributing to the differential analysis between clinical score and biomarker subgroups within this study. Future studies should therefore account for these variables in order to provide a more detailed calculation of time to administration. Thirdly, based on previous publications investigating the clinical utility of MR-proADM, additional secondary outcomes such as respiratory failure [49], acute kidney injury progression [50], and coronary ischaemia [25, 51] should be collected in order to further enhance our understanding of this novel biomarker due to its likely physiological action in endothelial injury and capillary leak. Finally, the relatively small number of patients in each subgroup, as well as low corresponding mortality and delayed ICU admission rates, allows for only initial hypotheses to be made and makes more detailed conclusions problematic. In addition, the presence of an independent validatory cohort, similar to that of Saeed et al. [4], utilising pre-specified and optimised cut-off values, would confer a greater degree of certainty to the obtained results.

## Conclusions

The use of MR-proADM upon ED presentation may aid in the identification of patients with low NEWS or qSOFA values at risk of a less intensive treatment and with a subsequently high likelihood of further disease progression, thus helping guide initial treatment decisions such as antibiotic administration and ICU admission as part of a multi-modal clinical assessment. An additional measurement at 72 h may facilitate the identification of patients with continuously low NEWS scores at risk of treatment failure and subsequent mortality. Nevertheless, these results should be confirmed in an interventional study setting before subsequent incorporation into clinical routine.

## Supplementary information

**Additional file 1: Table S1.** Initial clinical diagnoses and suspected infectious source. **Table S2.** Characteristics of patients hospitalised at 72 h. **Table S3.** Univariate Logistic regression for antibiotic administration (ED treatment). **Table S4.** Univariate Logistic regression for hospitalisation requirement (ED presentation). **Table S5**. Univariate Logistic regression for ICU admission prediction (ED presentation). **Table S6** Univariate Cox regression for the prediction of 28-day mortality (ED presentation). **Table S7**. Univariate Cox regression for the prediction of 28-day mortality (72 h). **Table S8.** Treatment and outcome in patients with low biomarker or score values (ED presentation). **Table S9.** Treatment and outcome in patients with high biomarker or score values (ED presentation). **Table S10.** Treatment and outcome in patients with low biomarker or score values (72 h). **Table S11.** Treatment and outcome in patients with high biomarker or score values (72 h). **Table S12.** Low NEWS patient subgroups classified according to Lactate and PCT kinetics between ED presentation and 72 h. **Table S13.** Patient subgroups stratified by lactate and MR-proADM. **Table S14.** Patient subgroups stratified by qSOFA and MR-proADM. **Table S15.** qSOFA and MR-proADM values upon presentation and 72 h within stratified subgroups. **Table S16.** Patient subgroups stratified by CRB-65 and MR-proADM. **Table S17.** Patient subgroups based on antibiotic administration using NEWS and MR-proADM. **Table S18.** Patient subgroups based on antibiotic administration using qSOFA and MR-proADM. **Figure S1**. AUROC analysis for antibiotic requirement during treatment within the ED. **Figure S2.** AUROC analysis for hospitalisation requirement upon ED presentation. **Figure S3.** AUROC analysis for ICU admission within 28 days of initial ED presentation. **Figure S4.** AUROC analysis for infection-related 28-day mortality upon ED presentation and 72 h.

## Data Availability

The datasets used and/or analysed during the present study are available from the corresponding author upon reasonable request.

## Abbreviations

AUC: Area under the curve
CAP: Community-acquired pneumonia
CI: Confidence interval
CRB-65: Severity scores for community-acquired pneumonia
CRP: C-reactive protein
ED: Emergency department
HR: Hazard ratio
IQR: Interquartile range
LRTI: Lower respiratory tract infection
MR-proADM: Mid-regional proadrenomedullin
*N*: Number
NEWS: National Early Warning Score
OR: Odds ratio
PCT: Procalcitonin
PSI: Pneumonia severity index
qSOFA: Quick Sequential Organ Failure Assessment
ROC: Receiver operating characteristic
RRT: Renal replacement therapy
SIRS: Systemic inflammatory response syndrome
SOFA: Sequential Organ Failure Assessment
UTI: Urinary tract infection
