# Supplementary material for: Biomarkers and clinical scores to identify patient populations at risk of delayed antibiotic administration or intensive care admission
Source: Crit Care. 2019 Oct 29;23:335. doi: 10.1186/s13054-019-2613-4 (PMC6819475; doi:10.1186/s13054-019-2613-4)
Supplement: Supplementary file 1 — Additional file 1: Table S1. Initial clinical diagnoses and suspected infectious source. Table S2. Characteristics of patients hospitalised at 72 h. Table S3. Univariate Logistic regression for antibiotic administration (ED treatment). Table S4. Univariate Logistic regression for hospitalisation requirement (ED presentation). Table S5. Univariate Logistic regression for ICU admission prediction (ED presentation). Table S6 Univariate Cox regression for the prediction of 28-day mortality (ED presentation). Table S7. Univariate Cox regression for the prediction of 28-day mortality (72 h). Table S8. Treatment and outcome in patients with low biomarker or score values (ED presentation). Table S9. Treatment and outcome in patients with high biomarker or score values (ED presentation). Table S10. Treatment and outcome in patients with low biomarker or score values (72 h). Table S11. Treatment and outcome in patients with high biomarker or score values (72 h). Table S12. Low NEWS patient subgroups classified according to Lactate and PCT kinetics between ED presentation and 72 h. Table S13. Patient subgroups stratified by lactate and MR-proADM. Table S14. Patient subgroups stratified by qSOFA and MR-proADM. Table S15. qSOFA and MR-proADM values upon presentation and 72 h within stratified subgroups. Table S16. Patient subgroups stratified by CRB-65 and MR-proADM. Table S17. Patient subgroups based on antibiotic administration using NEWS and MR-proADM. Table S18. Patient subgroups based on antibiotic administration using qSOFA and MR-proADM. Figure S1. AUROC analysis for antibiotic requirement during treatment within the ED. Figure S2. AUROC analysis for hospitalisation requirement upon ED presentation. Figure S3. AUROC analysis for ICU admission within 28 days of initial ED presentation. Figure S4. AUROC analysis for infection-related 28-day mortality upon ED presentation and 72 h. [file 13054_2019_2613_MOESM1_ESM.docx]

**Additional File 1**

**Biomarkers and clinical scores to identify patient populations at risk of delayed antibiotic administration or intensive care admission**

***Short title: Delayed antibiotic and ICU admission decisions in the ED***

**Juan González del Castillo MD PhD^1,2^, Darius Cameron Wilson PhD^3^, Carlota Clemente-Callejo MD^1^, Francisco Román MD^4^, Ignasi Bardés-Robles MD^5^, Inmaculada Jiménez MD^4^, Eva Orviz MD^6^, Macarena Dastis-Arias PhD^7^, Begoña Espinosa MD^4^, Fernando Tornero-Romero MD^6^, Jordi Giol-Amich MD PhD^5^, Veronica González MD^4^, and Ferran Llopis-Roca MD PhD^5^ on behalf of the INFURG-SEMES investigators**

1. Emergency Department. Hospital Clínico San Carlos. Madrid. Spain.
2. San Carlos Clinical Research Institute Hospital San Carlos (IdISSC), Madrid, Spain.
3. Shock, Organ Dysfunction and Resuscitation Research Group, Vall d’Hebron Institut of Research, Barcelona, Spain
4. Emergency Department, Short Stay Unit and Home Hospitalization Unit. Hospital General de Alicante. Alicante. Spain.
5. Emergency Department. Hospital Universitari de Bellvitge. Barcelona. Spain.
6. Internal Medicine Department. Hospital Clínico San Carlos. Madrid. Spain.
7. Clinical Laboratory Department. Hospital Universitari de Bellvitge. Barcelona. Spain.

**Corresponding author:** Dr. Juan González del Castillo, Emergency Department, Hospital Clinico Universitario San Carlos. Calle Profesor Martín Lagos, s/n. 28040 Madrid, Spain. Phone: +34 913303750. Email: [jgonzalezcast@gmail.com](mailto:jgonzalezcast@gmail.com)

**Contents**

**Supplementary Tables.....................................................................................................................3**

**Table S1. Initial clinical diagnoses and suspected infectious source**

**Table S2. Characteristics of patients hospitalised at 72 hours**

**Table S3. Univariate and Multivariate Logistic regression for antibiotic administration (ED treatment)**

**Table S4. Bivariate MR-proADM Logistic regression models for antibiotic administration (ED treatment)**

**Table S5. Univariate and Multivariate Logistic regression for hospitalisation requirement (ED presentation)**

**Table S6. Bivariate MR-proADM Logistic regression models for hospitalisation requirement (ED presentation)**

**Table S7. Univariate and Multivariate Logistic regression for ICU admission prediction (ED presentation)**

**Table S8. Bivariate MR-proADM Logistic regression models for ICU admission prediction (ED presentation)**

**Table S9. Univariate Cox regression for the prediction of 28-day mortality (ED presentation)**

**Table S10. Bivariate MR-proADM Logistic regression models for the prediction of 28-day mortality (ED presentation)**

**Table S11. Univariate Cox regression for the prediction of 28-day mortality (72 hours)**

**Table S12. Bivariate MR-proADM Logistic regression models for the prediction of 28-day mortality (72 hours)**

**Table S13. Treatment and outcome in patients with low biomarker or score values (ED presentation)**

**Table S14. Treatment and outcome in patients with high biomarker or score values (ED presentation)**

**Table S15. Treatment and outcome in patients with low biomarker or score values (72 hours)**

**Table S16. Treatment and outcome in patients with high biomarker or score values (72 hours)**

**Table S17. Patient subgroups stratified by lactate and MR-proADM**

**Table S18. Patient subgroups stratified by qSOFA and MR-proADM**

**Table S19. Patient subgroups stratified by CRB-65 and MR-proADM**

**Table S20. Patient subgroups based on antibiotic administration using NEWS and MR-proADM**

**Table S21. Patient subgroups based on antibiotic administration using qSOFA and MR-proADM**

**Table S22. Low NEWS patient subgroups classified according to Lactate and PCT kinetics between ED presentation and 72 hours**

**Table S23. qSOFA and MR-proADM values upon presentation and 72 hours within stratified subgroups**

**Supplementary Figures..................................................................................................................26**

**Figure S1. AUROC analysis for antibiotic requirement during treatment within the ED**

**Figure S2. AUROC analysis for hospitalisation requirement upon ED presentation**

**Figure S3. AUROC analysis for ICU admission within 28 days of initial ED presentation**

**Figure S4. AUROC analysis for infection-related 28-day mortality upon ED presentation and 72 hours**

**Supplementary Tables**

**Table S1. Initial clinical diagnoses and suspected infectious source**

| **Suspected source of infection** | **Patients**  **(*N*)** | **Hospital admission**  **(*N*, %)** | **ICU admission**  **(*N*, %)** | **28-day mortality**  ***(N*, %)** |
| --- | --- | --- | --- | --- |
| Bone and Joint | 4 | 3 (0.6%) | 1 (4.3%) | 1 (3.3%) |
| Cardiac | 2 | 1 (0.2%) | 0 (0.0%) | 1 (3.3%) |
| Intra-abdominal | 93 | 71 (14.3%) | 4 (17.4%) | 7 (23.3%) |
| Appendicitis | 6 |  |  |  |
| Cholangitis | 11 |  |  |  |
| Cholecystitis | 8 |  |  |  |
| Diverticulitis | 10 |  |  |  |
| Gastroenteritis | 17 |  |  |  |
| Other | 41 |  |  |  |
| Respiratory - lower | 220 | 185 (37.1%) | 8 (34.8%) | 14 (46.7%) |
| IECOPD | 8 |  |  |  |
| Pneumonia | 90 |  |  |  |
| Unspecified respiratory infection | 101 |  |  |  |
| Other | 21 |  |  |  |
| Respiratory - upper | 18 | 7 (1.4%) | 0 (0.0%) | 0 (0.0%) |
| Tonsillitis | 7 |  |  |  |
| Other | 11 |  |  |  |
| Skin and Soft Tissue | 36 | 25 (5.0%) | 2 (8.7%) | 1 (3.3%) |
| Cellulitis | 24 |  |  |  |
| Other | 12 |  |  |  |
| Surgical | 16 | 11 (2.2%) | 3 (13.0%) | 0 (0.0%) |
| Unknown origin | 81 | 47 (9.4%) | 1 (4.3%) | 1 (3.3%) |
| Urogenital | 214 | 148 (29.7%) | 4 (17.4%) | 5 (16.7%) |
| Pyelonephritis | 34 |  |  |  |
| Urinary tract infection | 112 |  |  |  |
| Other | 68 |  |  |  |

Percentages for each outcome per suspected source of infection, represent the proportion of the number of events per outcome within the total patient cohort. In brief, there were a total of *N*=498 hospitalisation, *N*=23 ICU admission and *N*=30 28-day mortality events. *N:* Number; *ICU:* Intensive Care Unit; *IECOPD*: Infective Exacerbation of Chronic Obstructive Pulmonary Disease.

**Table S2. Characteristics of patients hospitalised at 72 hours**

| **Patient Characteristics** | **Total cohort**  **(*N*=380)** |
| --- | --- |
| **Demographics** | |
| Age (years) (mean, SD) | 71.2 (16.7) |
| Male Sex (*N*, %) | 218 (57.4%) |
| **Disposition** | |
| Hospital admission (*N*, %) | 380 (100.0%) |
| Hospital length of stay (days) (median, Q1-Q3) | 7 [5 - 12] |
| ICU admission (*N*, %) | 7 (1.8%) |
| Infection-related 28-day mortality (*N*, %) | 26 (6.8%) |
| Hospital mortality (*N*, %) | 29 (7.6%) |
| **Sepsis classification** | |
| Sepsis-2 (*N*, %) | 211 (57.3%) |
| Sepsis-3 (*N*, %) | 229 (73.9%) |
| **Comorbidities** | |
| Cardiovascular disease (*N*, %) | 171 (45.0%) |
| Diabetes (*N*, %) | 103 (27.1%) |
| Immunodeficiency (*N*, %) | 65 (17.1%) |
| Liver disease (*N*, %) | 34 (8.9%) |
| Malignancy (*N*, %) | 115 (30.3%) |
| Neurological disorders (*N*, %) | 91 (23.9%) |
| Respiratory disease (*N*, %) | 119 (31.3%) |
| Renal disease (*N*, %) | 93 (24.5%) |
| **Infectious source** | |
| Bone and Joint (*N*, %) | 3 (0.8%) |
| Cardiac (*N*, %) | 1 (0.3%) |
| Intra-abdominal (*N*, %) | 57 (15.0%) |
| Respiratory - lower (*N*, %) | 148 (38.9%) |
| Respiratory - upper (*N*, %) | 1 (0.3%) |
| Skin and Soft Tissue (*N*, %) | 21 (5.5%) |
| Surgical-related (*N*, %) | 11 (2.9%) |
| Unknown origin (*N*, %) | 34 (8.9%) |
| Urogenital (*N*, %) | 104 (27.4%) |
| **Blood cultures** | |
| Blood cultures taken (*N*, %) | 252 (66.3%) |
| Positive blood cultures (*N*, %) | 49 (12.9%) |
| **Biomarkers and severity scores** | |
| MR-proADM (nmol/L) (median, Q1-Q3) | 1.08 [0.70 - 1.61] |
| PCT (ng/mL) (median, Q1-Q3) | 0.28 [0.12 - 0.89] |
| Lactate (mmol/L) (median, Q1-Q3) | 1.7 [1.3 - 2.2] |
| CRP (mg/L) (median, Q1-Q3) | 7.4 [3.07 - 17.43] |
| SOFA (points) (median, Q1-Q3) | 1 [0 - 1] |
| qSOFA (points) (median, Q1-Q3) | 2 [1 - 3] |
| SIRS (points) (median, Q1-Q3) | 1 [1 - 2] |
| NEWS (points) (median, Q1-Q3) | 0 [0 - 1] |
| CRB-65 (points) (median, Q1-Q3) | 2 [1 - 4] |

Values expressed in percentages (%) indicate the proportion of patients for each variable. Data are presented as mean (standard deviation, SD) or median [first quartile (Q1) - third quartile (Q3)] where specified. The chi-square (χ^2^) test was used to determine significance between the cohorts for categorical variables, Student’s t-test for the variable of age, and Mann-Whitney U test for hospitalisation duration, biomarker and clinical score variables. *CRB-65*: Severity score for community-acquired pneumonia; *CRP*: C-reactive protein; *ICU*: Intensive Care Unit; *MR-proADM*: Mid-regional proadrenomedullin; *N*: Number; *NEWS*: National Early Warning Score; *PCR*: Polymerase Chain Reaction; *PCT*: Procalcitonin; *qSOFA*: quick Sequential Organ Failure Assessment; *SIRS*: Systemic Inflammatory Response Syndrome; *SOFA*: Sequential Organ Failure Assessment.

**Table S3. Univariate and Multivariate Logistic regression for antibiotic administration (ED treatment)**

|  | **Biomarker or clinical score** | **Patients (*N*)** | **Antibiotics**  **(*N*)** | **LR χ²** | **p-value** | **C-index** | **OR [95% CI]** |
| --- | --- | --- | --- | --- | --- | --- | --- |
| Univariate | MR-proADM | 684 | 505 | 115.2 | <0.001 | 0.77 | 4.9 [3.5 - 6.8] |
|  | PCT | 684 | 505 | 80.6 | <0.001 | 0.73 | 3.5 [2.5 - 4.8] |
|  | Lactate | 533 | 412 | 1.8 | 0.176 | 0.52 | 1.2 [0.9 - 1.5] |
|  | CRP | 646 | 480 | 78.9 | <0.001 | 0.65 | 2.4 [1.7 - 3.3] |
|  | SOFA | 684 | 505 | 87.3 | <0.001 | 0.71 | 3.1 [2.4 - 4.0] |
|  | qSOFA | 684 | 505 | 51.4 | <0.001 | 0.65 | 3.5 [2.3 - 5.3] |
|  | NEWS | 684 | 505 | 103.4 | <0.001 | 0.74 | 5.5 [3.6 - 8.2] |
|  | CRB-65 | 684 | 505 | 66.8 | <0.001 | 0.70 | 2.9 [2.2 - 3.9] |
|  | SIRS | 684 | 505 | 61.6 | <0.001 | 0.67 | 2.1 [1.7 - 2.5] |
| Multivariate | MR-proADM | 684 | 505 | 133.1 | <0.001 | 0.79 | 3.5 [2.4 - 5.2] |
|  | PCT | 684 | 505 | 130.2 | <0.001 | 0.78 | 2.7 [1.9 - 3.7] |
|  | Lactate | 533 | 412 | 79.7 | <0.001 | 0.75 | 0.9 [0.7 - 1.2] |
|  | CRP | 646 | 480 | 145.4 | <0.001 | 0.78 | 2.5 [1.9 - 3.1] |
|  | SOFA | 684 | 505 | 107.8 | <0.001 | 0.79 | 2.9 [2.2 - 5.1] |
|  | qSOFA | 684 | 505 | 112.9 | <0.001 | 0.78 | 2.8 [1.8 - 4.4] |
|  | NEWS | 684 | 505 | 151.3 | <0.001 | 0.79 | 4.5 [2.9 - 6.9] |
|  | CRB-65 | 684 | 505 | 92.9 | <0.001 | 0.73 | 2.5 [1.3 - 4.6] |
|  | SIRS | 684 | 505 | 140.7 | <0.001 | 0.78 | 2.1 [1.7 - 2.6] |

*CI*: Confidence Interval; *CRB-65*: Severity score for community-acquired pneumonia; *CRP*: C-reactive protein; *LR*: Likelihood ratio; *MR-proADM*: Mid-regional proadrenomedullin; *N*: Number; *NEWS*: National Early Warning Score; *OR*: Odds Ratio; *PCT*: Procalcitonin; *qSOFA*: quick Sequential Organ Failure Assessment; *SIRS*: Systemic Inflammatory Response Syndrome; *SOFA*: Sequential Organ Failure Assessment.

**Table S4. Bivariate MR-proADM Logistic regression models for antibiotic administration (ED treatment)**

|  | **Biomarker or clinical score model + MR-proADM** | **Patients (*N*)** | **Antibiotics**  **(*N*)** | **LR χ²** | **p-value** | **C-index** | **OR [95% CI]** |
| --- | --- | --- | --- | --- | --- | --- | --- |
| Bivariate  MR-proADM model | PCT | 684 | 505 | 103.2 | <0.001 | 0.78 | 3.6 [2.5 – 5.2] |
|  | Lactate | 533 | 412 | 85.6 | <0.001 | 0.77 | 5.3 [3.5 – 7.9] |
|  | CRP | 646 | 480 | 160.2 | <0.001 | 0.81 | 4.4 [3.1 – 6.4] |
|  | SOFA | 684 | 505 | 129.1 | <0.001 | 0.78 | 3.4 [2.3 – 5.0] |
|  | qSOFA | 684 | 505 | 129.1 | <0.001 | 0.78 | 4.1 [2.9 – 5.7] |
|  | NEWS | 684 | 505 | 157.1 | <0.001 | 0.80 | 3.3 [2.3 – 4.7] |
|  | CRB-65 | 684 | 505 | 125.7 | <0.001 | 0.78 | 3.7 [2.6 – 5.3] |
|  | SIRS | 684 | 505 | 145.3 | <0.001 | 0.79 | 4.1 [2.9 – 5.8] |

*CI*: Confidence Interval; *CRB-65*: Severity score for community-acquired pneumonia; *CRP*: C-reactive protein; *LR*: Likelihood ratio; *MR-proADM*: Mid-regional proadrenomedullin; *N*: Number; *NEWS*: National Early Warning Score; *OR*: Odds Ratio; *PCT*: Procalcitonin; *qSOFA*: quick Sequential Organ Failure Assessment; *SIRS*: Systemic Inflammatory Response Syndrome; *SOFA*: Sequential Organ Failure Assessment.

**Table S5. Univariate and Multivariate Logistic regression for hospitalisation requirement (ED presentation)**

| **Biomarker or clinical score** | **Patients (*N*)** | **Hospitalisation**  **(*N*)** | **LR χ²** | **p-value** | **C-index** | **OR [95% CI]** |
| --- | --- | --- | --- | --- | --- | --- |
| MR-proADM | 684 | 498 | 151.2 | <0.001 | 0.82 | 6.6 [4.6 - 9.4] |
| PCT | 684 | 498 | 78.0 | <0.001 | 0.73 | 3.3 [2.4 - 4.5] |
| Lactate | 533 | 420 | 2.7 | 0.098 | 0.53 | 1.2 [1.0 - 1.6] |
| CRP | 646 | 479 | 45.0 | <0.001 | 0.66 | 2.0 [1.6 - 2.5] |
| SOFA | 684 | 498 | 105.2 | <0.001 | 0.75 | 5.7 [3.7 - 9.4] |
| qSOFA | 684 | 498 | 62.2 | <0.001 | 0.65 | 4.1 [2.7 - 6.3] |
| NEWS | 684 | 498 | 114.5 | <0.001 | 0.75 | 6.0 [4.0 - 9.1] |
| CRB-65 | 684 | 498 | 74.0 | <0.001 | 0.69 | 2.5 [1.9 - 2.6] |
| SIRS | 684 | 498 | 58.6 | <0.001 | 0.68 | 2.0 [1.7 - 2.4] |
| MR-proADM | 684 | 498 | 169.7 | <0.001 | 0.81 | 5.2 [3.4 - 7.8] |
| PCT | 684 | 498 | 129.3 | <0.001 | 0.77 | 2.6 [1.9 - 3.6] |
| Lactate | 533 | 420 | 67.9 | <0.001 | 0.74 | 1.0 [0.7 - 1.3] |
| CRP | 646 | 479 | 114.1 | <0.001 | 0.76 | 1.9 [1.5 - 2.4] |
| SOFA | 684 | 498 | 132.2 | <0.001 | 0.77 | 3.8 [2.5 - 6.0] |
| qSOFA | 684 | 498 | 122.5 | <0.001 | 0.74 | 3.4 [2.2 - 5.4] |
| NEWS | 684 | 498 | 162.6 | <0.001 | 0.80 | 5.1 [3.3 - 8.0] |
| CRB-65 | 684 | 498 | 96.4 | <0.001 | 0.74 | 3.0 [1.6 - 5.6] |
| SIRS | 684 | 498 | 140.2 | <0.001 | 0.78 | 2.1 [1.7 - 2.6] |

*CI*: Confidence Interval; *CRB-65*: Severity score for community-acquired pneumonia; *CRP*: C-reactive protein; *LR*: Likelihood ratio; *MR-proADM*: Mid-regional proadrenomedullin; *N*: Number; *NEWS*: National Early Warning Score; *OR*: Odds Ratio; *PCT*: Procalcitonin; *qSOFA*: quick Sequential Organ Failure Assessment; *SIRS*: Systemic Inflammatory Response Syndrome; *SOFA*: Sequential Organ Failure Assessment.

**Table S6. Bivariate MR-proADM Logistic regression models for hospitalisation requirement (ED presentation)**

|  | **Biomarker or clinical score model + MR-proADM** | **Patients (*N*)** | **Hospitalisation**  **(*N*)** | **LR χ²** | **p-value** | **C-index** | **OR [95% CI]** |
| --- | --- | --- | --- | --- | --- | --- | --- |
| Bivariate  MR-proADM model | PCT | 684 | 498 | 159.4 | <0.001 | 0.80 | 5.2 [3.5 - 7.7] |
|  | Lactate | 533 | 420 | 94.7 | <0.001 | 0.78 | 6.3 [4.0 - 9.7] |
|  | CRP | 646 | 479 | 157.0 | <0.001 | 0.81 | 5.9 [4.0 - 8.8] |
|  | SOFA | 684 | 498 | 165.8 | <0.001 | 0.81 | 4.5 [3.0 - 6.8] |
|  | qSOFA | 684 | 498 | 167.7 | <0.001 | 0.80 | 5.4 [3.7 - 7.9] |
|  | NEWS | 684 | 498 | 193.1 | <0.001 | 0.82 | 4.5 [3.1 - 6.6] |
|  | CRB-65 | 684 | 498 | 159.4 | <0.001 | 0.80 | 5.1 [3.4 - 7.6] |
|  | SIRS | 684 | 498 | 175.9 | <0.001 | 0.81 | 5.7 [3.9 - 8.2] |

*CI*: Confidence Interval; *CRB-65*: Severity score for community-acquired pneumonia; *CRP*: C-reactive protein; *LR*: Likelihood ratio; *MR-proADM*: Mid-regional proadrenomedullin; *N*: Number; *NEWS*: National Early Warning Score; *OR*: Odds Ratio; *PCT*: Procalcitonin; *qSOFA*: quick Sequential Organ Failure Assessment; *SIRS*: Systemic Inflammatory Response Syndrome; *SOFA*: Sequential Organ Failure Assessment.

**Table S7. Univariate and Multivariate Logistic regression for ICU admission prediction (ED presentation)**

| **Biomarker or clinical score** | **Patients (*N*)** | **ICU admission**  **(*N*)** | **LR χ²** | **p-value** | **C-index** | **OR [95% CI]** |
| --- | --- | --- | --- | --- | --- | --- |
| MR-proADM | 684 | 23 | 25.4 | <0.001 | 0.79 | 4.1 [2.3 - 7.1] |
| PCT | 684 | 23 | 12.5 | <0.001 | 0.71 | 2.2 [1.5 - 3.4] |
| Lactate | 533 | 22 | 0.7 | 0.408 | 0.58 | 1.2 [ 0.8 - 1.9] |
| CRP | 646 | 23 | 7.6 | 0.006 | 0.67 | 2.1 [1.2 - 3.6] |
| SOFA | 684 | 23 | 7.4 | 0.006 | 0.70 | 2.2 [1.3 - 3.8] |
| qSOFA | 684 | 23 | 7.5 | 0.006 | 0.62 | 2.1 [1.3 - 3.4] |
| NEWS | 684 | 23 | 8.5 | 0.004 | 0.62 | 2.0 [1.3 - 3.2] |
| CRB-65 | 684 | 23 | 5.3 | 0.021 | 0.63 | 3.0 [1.2 - 7.5] |
| SIRS | 684 | 23 | 0.1 | 0.725 | 0.52 | 1.1 [0.7 - 1.6] |
| MR-proADM | 684 | 23 | 34.0 | <0.001 | 0.82 | 5.8 [3.1 - 10.8] |
| PCT | 684 | 23 | 14.8 | 0.022 | 0.74 | 2.2 [1.4 - 3.4] |
| Lactate | 533 | 22 | 4.0 | 0.673 | 0.60 | 1.3 [0.8 - 2.0] |
| CRP | 646 | 23 | 10.2 | 0.115 | 0.71 | 2.0 [1.2 - 3.5] |
| SOFA | 684 | 23 | 9.7 | 0.136 | 0.75 | 2.3 [1.3 - 4.2] |
| qSOFA | 684 | 23 | 10.5 | 0.105 | 0.66 | 2.2 [1.3 - 3.8] |
| NEWS | 684 | 23 | 12.4 | 0.053 | 0.65 | 2.2 [1.4 - 3.6] |
| CRB-65 | 684 | 23 | 11.2 | 0.083 | 0.67 | 1.7 [1.2 - 2.6] |
| SIRS | 684 | 23 | 3.2 | 0.785 | 0.60 | 1.1 [0.7 - 1.6] |

Patients admitted to the ICU after 28 days following initial ED presentation were not treated as an ICU positive event. *CI*: Confidence Interval; *CRB-65*: Severity score for community-acquired pneumonia; *CRP*: C-reactive protein; *LR*: Likelihood ratio; *MR-proADM*: Mid-regional proadrenomedullin; *N*: Number; *NEWS*: National Early Warning Score; *OR*: Odds Ratio; *PCT*: Procalcitonin; *qSOFA*: quick Sequential Organ Failure Assessment; *SIRS*: Systemic Inflammatory Response Syndrome; *SOFA*: Sequential Organ Failure Assessment.

**Table S8. Bivariate MR-proADM Logistic regression models for ICU admission prediction (ED presentation)**

|  | **Biomarker or clinical score model + MR-proADM** | **Patients (*N*)** | **ICU admission**  **(*N*)** | **LR χ²** | **p-value** | **C-index** | **OR [95% CI]** |
| --- | --- | --- | --- | --- | --- | --- | --- |
| Bivariate  MR-proADM model | PCT | 684 | 23 | 25.4 | <0.001 | 0.79 | 3.8 [1.7 - 8.4] |
|  | Lactate | 533 | 22 | 18.4 | <0.001 | 0.76 | 3.6 [2.0 - 6.8] |
|  | CRP | 646 | 23 | 28.9 | <0.001 | 0.81 | 3.7 [2.1 - 6.4] |
|  | SOFA | 684 | 23 | 25.6 | <0.001 | 0.79 | 4.5 [2.2 - 9.3] |
|  | qSOFA | 684 | 23 | 25.8 | <0.001 | 0.79 | 3.7 [2.0 - 6.9] |
|  | NEWS | 684 | 23 | 26.1 | <0.001 | 0.78 | 3.7 [2.0 - 6.7] |
|  | CRB-65 | 684 | 23 | 25.4 | <0.001 | 0.79 | 4.1 [2.2 - 7.7] |
|  | SIRS | 684 | 23 | 26.5 | <0.001 | 0.80 | 4.4 [2.4 - 7.8] |

*CI*: Confidence Interval; *CRB-65*: Severity score for community-acquired pneumonia; *CRP*: C-reactive protein; *LR*: Likelihood ratio; *MR-proADM*: Mid-regional proadrenomedullin; *N*: Number; *NEWS*: National Early Warning Score; *OR*: Odds Ratio; *PCT*: Procalcitonin; *qSOFA*: quick Sequential Organ Failure Assessment; *SIRS*: Systemic Inflammatory Response Syndrome; *SOFA*: Sequential Organ Failure Assessment.

**Table S9.** **Univariate Cox regression for the prediction of 28-day mortality (ED presentation)**

| **Biomarker or clinical score** | **Patients (*N*)** | **Mortality**  **(*N*)** | **LR χ²** | **p-value** | **C-index** | **HR [95% CI]** |
| --- | --- | --- | --- | --- | --- | --- |
| MR-proADM | 684 | 30 | 36.6 | <0.001 | 0.84 | 4.1 [2.6 - 6.5] |
| PCT | 684 | 30 | 10.9 | <0.001 | 0.68 | 1.9 [1.3 - 2.7] |
| Lactate | 533 | 30 | 6.9 | <0.01 | 0.67 | 1.6 [1.2 - 2.2] |
| CRP | 646 | 28 | 0.0 | 0.994 | 0.50 | 1.0 [0.7 - 1.5] |
| SOFA | 684 | 30 | 36.2 | <0.001 | 0.84 | 3.9 [2.6 - 5.7] |
| qSOFA | 684 | 30 | 25.8 | <0.001 | 0.86 | 2.9 [2.0 - 4.2] |
| NEWS | 684 | 30 | 18.6 | <0.001 | 0.77 | 2.4 [1.7 - 3.4] |
| CRB-65 | 684 | 30 | 25.3 | <0.001 | 0.82 | 2.7 [1.9 - 4.0] |
| SIRS | 684 | 30 | 5.1 | 0.025 | 0.66 | 1.5 [1.1 - 2.0] |

*CI*: Confidence Interval; *CRB-65*: Severity score for community-acquired pneumonia; *CRP*: C-reactive protein; *HR*: Hazard Ratio; *LR*: Likelihood ratio; *MR-proADM*: Mid-regional proadrenomedullin; *N*: Number; *NEWS*: National Early Warning Score; *PCT*: Procalcitonin; *qSOFA*: quick Sequential Organ Failure Assessment; *SIRS*: Systemic Inflammatory Response Syndrome; *SOFA*: Sequential Organ Failure Assessment.

**Table S10. Bivariate MR-proADM Logistic regression models for the prediction of 28-day mortality (ED presentation)**

|  | **Biomarker or clinical score model + MR-proADM** | **Patients (*N*)** | **Mortality**  **(*N*)** | **LR χ²** | **p-value** | **HR [95% CI]** |
| --- | --- | --- | --- | --- | --- | --- |
| Bivariate  MR-proADM model | PCT | 684 | 30 | 38.1 | <0.001 | 5.7 [2.8 - 11.6] |
|  | Lactate | 533 | 30 | 30.4 | <0.001 | 3.3 [2.0 - 5.5] |
|  | CRP | 646 | 28 | 32.7 | <0.001 | 4.1 [2.5 - 6.6] |
|  | SOFA | 684 | 30 | 46.1 | <0.001 | 2.4 [1.4 - 4.2] |
|  | qSOFA | 684 | 30 | 43.6 | <0.001 | 3.0 [1.8 - 5.1] |
|  | NEWS | 684 | 30 | 40.3 | <0.001 | 3.4 [2.0 - 5.6] |
|  | CRB-65 | 684 | 30 | 42.1 | <0.001 | 3.0 [1.8 - 5.2] |
|  | SIRS | 684 | 30 | 37.1 | <0.001 | 3.9 [2.4 - 6.3] |

*CI*: Confidence Interval; *CRB-65*: Severity score for community-acquired pneumonia; *CRP*: C-reactive protein; *LR*: Likelihood ratio; *MR-proADM*: Mid-regional proadrenomedullin; *N*: Number; *NEWS*: National Early Warning Score; *OR*: Odds Ratio; *PCT*: Procalcitonin; *qSOFA*: quick Sequential Organ Failure Assessment; *SIRS*: Systemic Inflammatory Response Syndrome; *SOFA*: Sequential Organ Failure Assessment.

**Table S11. Univariate Cox regression for the prediction of 28-day mortality (72 hours)**

| **Biomarker or clinical score** | **Patients (*N*)** | **Mortality**  **(*N*)** | **LR χ²** | **p-value** | **C-index** | **HR [95% CI]** |
| --- | --- | --- | --- | --- | --- | --- |
| MR-proADM | 375 | 25 | 20.7 | <0.001 | 0.75 | 3.0 [1.9 - 4.7] |
| PCT | 370 | 25 | 5.9 | 0.015 | 0.62 | 1.6 [1.1 - 2.4] |
| Lactate | 266 | 24 | 0.0 | 0.908 | 0.59 | 1.0 [0.7 - 1.4] |
| CRP | 281 | 20 | 1.3 | 0.256 | 0.55 | 0.7 [0.5 - 1.2] |
| SOFA | 375 | 25 | 19.8 | <0.001 | 0.75 | 2.3 [1.7 - 3.2] |
| qSOFA | 376 | 25 | 3.9 | 0.049 | 0.68 | 1.7 [1.0 - 2.8] |
| NEWS | 376 | 25 | 8.8 | 0.003 | 0.68 | 1.8 [1.3 - 2.5] |
| CRB-65 | 376 | 25 | 7.1 | 0.008 | 0.71 | 1.8 [1.2 - 2.8] |
| SIRS | 376 | 25 | 4.6 | 0.031 | 0.67 | 1.6 [1.1 - 2.8] |

*CI*: Confidence Interval; *CRB-65*: Severity score for community-acquired pneumonia; *CRP*: C-reactive protein; *HR*: Hazard Ratio; *LR*: Likelihood ratio; *MR-proADM*: Mid-regional proadrenomedullin; *N*: Number; *NEWS*: National Early Warning Score; *PCT*: Procalcitonin; *qSOFA*: quick Sequential Organ Failure Assessment; *SIRS*: Systemic Inflammatory Response Syndrome; *SOFA*: Sequential Organ Failure Assessment.

**Table S12. Bivariate MR-proADM Logistic regression models for the prediction of 28-day mortality (72 hours)**

|  | **Biomarker or clinical score model + MR-proADM** | **Patients (*N*)** | **Mortality**  **(*N*)** | **LR χ²** | **p-value** | **HR [95% CI]** |
| --- | --- | --- | --- | --- | --- | --- |
| Bivariate  MR-proADM model | PCT | 375 | 25 | 21.9 | <0.001 | 3.2 [1.8 - 5.7] |
|  | Lactate | 370 | 25 | 13.4 | 0.001 | 2.5 [1.6 - 4.1] |
|  | CRP | 266 | 24 | 17.4 | <0.001 | 3.1 [1.8 - 5.3] |
|  | SOFA | 281 | 20 | 27.0 | <0.001 | 2.1 [1.2 - 3.6] |
|  | qSOFA | 375 | 25 | 21.8 | <0.001 | 2.8 [1.8 - 4.6] |
|  | NEWS | 376 | 25 | 24.7 | <0.001 | 2.7 [1.7 - 4.3] |
|  | CRB-65 | 376 | 25 | 23.1 | <0.001 | 2.8 [1.7 - 4.5] |
|  | SIRS | 376 | 25 | 22.7 | <0.001 | 2.8 [1.7 - 4.4] |

*CI*: Confidence Interval; *CRB-65*: Severity score for community-acquired pneumonia; *CRP*: C-reactive protein; *LR*: Likelihood ratio; *MR-proADM*: Mid-regional proadrenomedullin; *N*: Number; *NEWS*: National Early Warning Score; *OR*: Odds Ratio; *PCT*: Procalcitonin; *qSOFA*: quick Sequential Organ Failure Assessment; *SIRS*: Systemic Inflammatory Response Syndrome; *SOFA*: Sequential Organ Failure Assessment.

**Table S13. Treatment and outcome in patients with low biomarker or clinical score values (ED presentation)**

| **Biomarker**  **or score** | **Selected**  **cut-offs** | **Population**  **(*N*)** | **Antibiotic administration**  ***N* (%)** | **Mortality**  ***N* (%)** | **ICU admission**  ***N* (%)** | **Hospitalisation**  ***N* (%)** | **Length of hospitalisation**  **(days)** | **Infection related readmission**  ***N* (%)** |
| --- | --- | --- | --- | --- | --- | --- | --- | --- |
| MR-proADM | <1.54 nmol/L* | 475 | 313 (65.9%) | 4 (0.8%) | 8 (1.7%) | 307 (64.0%) | 2 [0 - 6] | 26 (5.5%) |
| MR-proADM | <1.77 nmol/L† | 525 | 360 (68.6%) | 6 (1.1%) | 9 (1.7%) | 353 (67.2%) | 2 [0 - 6] | 28 (5.3%) |
| PCT | <0.20 ng/mL† | 330 | 200 (60.6%) | 6 (1.8%) | 4 (1.2%) | 193 (58.5%) | 1 [0 - 6] | 20 (6.6%) |
| PCT | <0.25 ng/mL* | 369 | 231 (62.6%) | 8 (2.2%) | 5 (1.4%) | 224 (60.7%) | 2 [0 - 6] | 21 (5.7%) |
| Lactate | <1.90 mmol/L† | 355 | 271 (76.3%) | 9 (2.5%) | 11 (3.1%) | 272 (76.6%) | 4 [0 - 7] | 27 (7.6%) |
| Lactate | <2.0 mmol/L* | 371 | 284 (76.5%) | 13 (3.5%) | 13 (3.5%) | 286 (77.1%) | 4 [0 - 7] | 28 (7.5%) |
| CRP | <7.0 mg/L† | 251 | 148 (59.0%) | 7 (2.8%) | 3 (1.2%) | 155 (61.8%) | 3 [0 - 7] | 19 (7.6%) |
| SOFA | <2 points* | 376 | 233 (62.0%) | 2 (0.5%) | 6 (1.6%) | 222 (59.0%) | 2 [0 - 6] | 24 (6.4%) |
| SOFA | <3 points† | 491 | 332 (67.6%) | 7 (1.4%) | 12 (2.4%) | 324 (66.0%) | 3 [0 - 7] | 33 (6.7%) |
| qSOFA | <1 point† | 452 | 300 (66.4%) | 5 (0.9%) | 10 (2.2%) | 291 (64.4%) | 2 [0 - 7] | 36 (8.0%) |
| qSOFA | <2 points* | 637 | 458 (71.9%) | 23 (5.0%) | 19 (3.0%) | 451 (70.8%) | 3 [0 - 8] | 48 (7.5%) |
| NEWS | <4 points* | 427 | 271 (63.5%) | 8 (1.9%) | 9 (2.1%) | 261 (61.1%) | 2 [0 - 6] | 35 (8.2%) |
| NEWS | <5 points† | 499 | 329 (65.9%) | 10 (2.0%) | 13 (2.6%) | 323 (64.7%) | 2 [0 - 6] | 39 (7.8%) |
| CRB-65 | <2 points** | 502 | 345 (68.7%) | 11 (2.2%) | 12 (2.4%) | 332 (66.1%) | 2 [0 - 7] | 40 (8.0%) |
| SIRS | <2 points** | 375 | 238 (63.5%) | 11 (2.9%) | 12 (3.2%) | 236 (62.9%) | 2 [0 - 6] | 30 (8.0%) |

* denotes the use of a pre-established cut-off, † donates use of the optimised cut-off, and ** indicates that both the pre-established and optimised cut-offs were identical. *CI*: Confidence interval; *CRB-65*: Severity score for community-acquired pneumonia; *CRP*: C-reactive protein; ICU: Intensive Care Unit; MR*-proADM*: Mid-regional proadrenomedullin; *N*: Number; *NEWS*: National Early Warning Score; *PCT*: Procalcitonin; *qSOFA*: quick Sequential Organ Failure Assessment; *SOFA*: Sequential Organ Failure Assessment.

**Table S14. Treatment and outcome in patients with high biomarker or clinical score values (ED presentation)**

| **Biomarker**  **or score** | **Selected**  **cut-offs** | **Population**  **(*N*)** | **Antibiotic administration**  ***N* (%)** | **Mortality**  ***N* (%)** | **ICU admission**  ***N* (%)** | **Hospitalisation**  **N (%)** | **Length of hospitalisation**  **(days)** | **Infection related readmission**  ***N* (%)** |
| --- | --- | --- | --- | --- | --- | --- | --- | --- |
| MR-proADM | ≥1.54 nmol/L* | 209 | 192 (91.9%) | 26 (12.4%) | 15 (7.2%) | 191 (91.4%) | 7 [3 - 12] | 25 (12.0%) |
| MR-proADM | ≥1.77 nmol/L† | 159 | 145 (91.2%) | 24 (15.1%) | 14 (8.8%) | 145 (91.2%) | 7 [4 - 13] | 23 (14.5%) |
| PCT | ≥0.20 ng/mL† | 354 | 305 (86.2%) | 24 (6.8%) | 19 (5.4%) | 305 (86.2%) | 5 [2 - 10] | 31 (8.9%) |
| PCT | ≥0.25 ng/mL* | 315 | 274 (87.0%) | 22 (7.0%) | 18 (5.7%) | 274 (87.0%) | 5 [2 - 10] | 30 (9.5%) |
| Lactate | ≥1.90 mmol/L† | 178 | 141 (79.2%) | 21 (11.8%) | 11 (6.2%) | 148 (83.1%) | 5 [2 - 10] | 12 (6.7%) |
| Lactate | ≥2.0 mmol/L* | 162 | 128 (79.0%) | 17 (10.5%) | 9 (5.6%) | 134 (82.7%) | 5 [1.5 - 10] | 11 (6.8%) |
| CRP | ≥7.0 mg/L† | 395 | 332 (84.0%) | 21 (5.3%) | 20 (5.1%) | 324 (82.0%) | 4 [2 - 8] | 31 (7.8%) |
| SOFA | ≥2 points* | 308 | 273 (88.6%) | 28 (9.1%) | 17 (5.5%) | 276 (89.6%) | 6 [3 - 10] | 27 (8.8%) |
| SOFA | ≥3 points† | 193 | 173 (89.6%) | 23 (11.9%) | 11 (5.7%) | 174 (90.2%) | 7 [4 - 11] | 18 (10.4%) |
| qSOFA | ≥1 point† | 232 | 205 (88.4%) | 25 (12.2%) | 13 (5.6%) | 207 (89.2%) | 6 [3 - 11] | 15 (6.5%) |
| qSOFA | ≥2 points* | 47 | 47 (100.0%) | 7 (14.9%) | 4 (8.5%) | 47 (100.0%) | 7 [4 - 13] | 0 (0.0%) |
| NEWS | ≥4 points* | 257 | 234 (91.1%) | 22 (8.6%) | 14 (5.4%) | 237 (92.2%) | 6 [3 - 11] | 16 (6.2%) |
| NEWS | ≥5 points† | 185 | 176 (95.1%) | 20 (10.8%) | 10 (5.4%) | 175 (94.6%) | 7 [4 - 12] | 12 (6.5%) |
| CRB-65 | ≥2 points** | 182 | 160 (87.9%) | 19 (10.4%) | 11 (6.0%) | 166 (91.2%) | 7 [3 - 12] | 11 (6.0%) |
| SIRS | ≥2 points** | 309 | 267 (86.4%) | 19 (6.1%) | 11 (3.6%) | 262 (84.8%) | 5 [2 - 10] | 21 (6.8%) |

* denotes the use of a pre-established cut-off, † donates use of the optimised cut-off, and ** indicates that both the pre-established and optimised cut-offs were identical. *CI*: Confidence interval; *CRB-65*: Severity score for community-acquired pneumonia; *CRP*: C-reactive protein; *ICU*: Intensive Care Unit; *MR-proADM*: Mid-regional proadrenomedullin; *N*: Number; *NEWS*: National Early Warning Score; *PCT*: Procalcitonin; *qSOFA*: quick Sequential Organ Failure Assessment; *SOFA*: Sequential Organ Failure Assessment.

**Table S15. Treatment and outcome in patients with low biomarker or score values (72 hours)**

| **Biomarker**  **or score** | **Selected**  **cut-offs** | **Population**  **(*N*)** | **Mortality**  ***N* (%)** | **ICU admission**  ***N* (%)** | **Length of hospitalisation**  **(days)** | **Infection related readmission**  ***N* (%)** |
| --- | --- | --- | --- | --- | --- | --- |
| MR-proADM | <1.57 nmol/L† | 273 | 8 (2.9%) | 3 (1.1%) | 7 [5 - 10] | 22 (8.1%) |
| PCT | <1.78 ng/mL† | 296 | 13 (4.3%) | 6 (2.0%) | 7 [5 - 11] | 24 (8.1%) |
| Lactate | <1.7 mmol/L† | 121 | 6 (5.0%) | 2 (1.7%) | 7 [5 - 13] | 11 (9.1%) |
| CRP | <16.4 mg/L† | 202 | 14 (6.9%) | 3 (1.5%) | 7 [4 - 10] | 15 (7.4%) |
| SOFA | <2 points† | 183 | 3 (1.6%) | 3 (1.6%) | 7 [5 - 12] | 25 (13.7%) |
| qSOFA | <1 point† | 351 | 22 (6.3%) | 7 (2.0%) | 7 [4- 1 2] | 29 (8.3%) |
| NEWS | <4 points† | 268 | 11 (4.1%) | 7 (2.6%) | 7 [4.25 - 12.75] | 23 (8.6%) |
| CRB-65 | <1 point† | 90 | 1 (1.1%) | 1 (1.1%) | 7 [5 - 11] | 12 (13.3%) |
| SIRS | <1 point† | 158 | 7 (4.4%) | 5 (3.2%) | 6 [4 - 11] | 14 (8.9%) |

† donates use of the optimised cut-off. *CRB-65*: Severity score for community-acquired pneumonia; *CRP*: C-reactive protein; *ICU*: Intensive Care Unit; *MR-proADM*: Mid-regional proadrenomedullin; *N*: Number; *NEWS*: National Early Warning Score; *PCT*: Procalcitonin; *qSOFA*: quick Sequential Organ Failure Assessment; *SOFA*: Sequential Organ Failure Assessment.

**Table S16. Treatment and outcome in patients with high biomarker or score values (72 hours)**

| **Biomarker**  **or score** | **Selected**  **cut-offs** | **Population**  **(*N*)** | **Mortality**  ***N* (%)** | **ICU admission**  ***N* (%)** | **Length of hospitalisation**  **(days)** | **Infection related readmission**  ***N* (%)** |
| --- | --- | --- | --- | --- | --- | --- |
| MR-proADM | ≥1.57 nmol/L† | 102 | 17 (16.7%) | 4 (3.9%) | 9.5 [6 - 19.25] | 9 (8.8%) |
| PCT | ≥1.78 ng/mL† | 74 | 12 (16.2%) | 1 (1.4%) | 8.5 [6 - 13.75] | 6 (8.1%) |
| Lactate | ≥1.7 mmol/L† | 145 | 18 (12.4%) | 5 (3.4%) | 8 [4 - 10] | 12 (8.3%) |
| CRP | ≥16.4 mg/L† | 79 | 6 (7.6%) | 0 (0.0%) | 7.5 [5 - 14] | 7 (8.9%) |
| SOFA | ≥2 points† | 192 | 22 (11.5%) | 4 (2.1%) | 8 [5 - 14] | 18 (9.4%) |
| qSOFA | ≥1 point† | 119 | 13 (10.9%) | 0 (0.0%) | 8 [6 - 14] | 9 (7.6%) |
| NEWS | ≥4 points† | 108 | 14 (13.0%) | 0 (0.0%) | 9 [6 - 14] | 8 (7.4%) |
| CRB-65 | ≥1 point† | 286 | 24 (8.4%) | 6 (2.1%) | 7 [5 - 13] | 20 (7.0%) |
| SIRS | ≥1 point† | 218 | 18 (8.3%) | 2 (0.9%) | 8 [5 - 14] | 17 (7.8%) |

† donates use of the optimised cut-off. *CRB-65*: Severity score for community-acquired pneumonia; *CRP*: C-reactive protein; *ICU*: Intensive Care Unit; *MR-proADM*: Mid-regional proadrenomedullin; *N*: Number; *NEWS*: National Early Warning Score; *PCT*: Procalcitonin; *qSOFA*: quick Sequential Organ Failure Assessment; *SOFA*: Sequential Organ Failure Assessment.

**Table S17. Patient subgroups stratified by lactate and MR-proADM**

|  | Patient populations stratified by Lactate and MR-proADM | | | | | | | |
| --- | --- | --- | --- | --- | --- | --- | --- | --- |
| **Patient subgroups** | MR-proADM  (nmol/L) | Lactate  (mmol/L) | MR-proADM  (nmol/L) | Lactate  (mmol/L) | MR-proADM  (nmol/L) | Lactate  (mmol/L) | MR-proADM  (nmol/L) | Lactate  (mmol/L) |
|  | <1.77 | <2.0 | ≥1.77 | <2.0 | <1.77 | ≥2.0 | ≥1.77 | ≥2.0 |
| **Population** *N* (%) | 296 (55.5%) | | 75 (14.1%) | | 98 (18.4%) | | 64 (12.0%) | |
| **Antibiotic administration** *N* (%) | 214 (72.3%) | | 70 (94.7%) | | 71 (72.4%) | | 57 (89.1%) | |
| **Time to antibiotic administration** (mins) (median [Q1-Q4]) | 182 [90 - 329] | | 180 [116 - 300] | | 120 [56 - 240] | | 120 [60 - 203] | |
| **Antibiotic administration <180 mins** *N* (%) | 86 (40.2%) | | 32 (45.7%) | | 44 (62.0%) | | 38 (66.7%) | |
| **Hospitalisation** *N* (%) | 217 (73.3%) | | 69 (92.0%) | | 76 (77.6%) | | 58 (90.6%) | |
| **Length of hospitalisation** (days) (median [Q1-Q4]) | 2 [0 - 7] | | 7 [4 - 13.5] | | 3 [0 - 7.5] | | 7 [4 - 13] | |
| **Infection related readmission** *N* (%) | 17 (5.7%) | | 11 (14.7%) | | 3 (3.1%) | | 8 (12.5%) | |
| **ICU admission** *N* (%) | 7 (2.4%) | | 6 (8.0%) | | 2 (2.0%) | | 7 (10.9%) | |
| **Time to ICU admission** (days) (median [Q1-Q4]) | 13 [2 - 17] | | 1 [0 - 4.75] | | NA | | 0 [0 - 0] | |
| **Number of immediate ICU admissions** *N* (%) | 1 (4.5%) | | 3 (13.6%) | | 1 (4.5%) | | 4 (18.2%) | |
| **Number of delayed ICU admissions** *N* (%) | 1 (4.5%) | | 3 (13.6%) | | 1 (4.5%) | | 2 (9.1%) | |
| **Number of late ICU admissions** *N* (%) | 5 (22.7%) | | 0 (0.0%) | | 0 (0.0%) | | 1 (4.5%) | |
| **Infection-related 28-day mortality** *N* (%) | 3 (1.0%) | | 10 (13.3%) | | 3 (3.1%) | | 14 (21.9%) | |
| **Hospital mortality** *N* (%) | 4 (1.4%) | | 10 (13.3%) | | 6 (6.1%) | | 14 (21.9%) | |
| **Disease progression** *N* (%) | 24 (8.1%) | | 20 (26.7%) | | 8 (8.2%) | | 24 (37.5%) | |

Number of immediate, delayed or late ICU admissions are expressed as a percentage of the total number of ICU admissions. *ICU*: Intensive Care Unit; *MR-proADM*: Mid-regional proadrenomedullin; *N*: Number.

**Table S18. Patient subgroups stratified by qSOFA and MR-proADM**

|  | Patient populations stratified by qSOFA and MR-proADM | | | | | | | |
| --- | --- | --- | --- | --- | --- | --- | --- | --- |
| **Patient subgroups** | MR-proADM  (nmol/L) | qSOFA  (points) | MR-proADM  (nmol/L) | qSOFA  (points) | MR-proADM  (nmol/L) | qSOFA  (points) | MR-proADM  (nmol/L) | qSOFA  (points) |
|  | <1.77 | <2 | ≥1.77 | <2 | <1.77 | ≥2 | ≥1.77 | ≥2 |
| **Population** *N* (%) | 506 (74.0%) | | 131 (19.2%) | | 19 (2.8%) | | 28 (4.1%) | |
| **Antibiotic administration** *N* (%) | 341 (67.4%) | | 117 (89.3%) | | 19 (100.0%) | | 28 (100.0%) | |
| **Time to antibiotic administration** (mins) (median [Q1-Q4]) | 210 [120 - 360] | | 210 [120 - 360] | | 180 [120 - 234] | | 120 [85 - 221] | |
| **Antibiotic administration <180 mins** *N* (%) | 132 (38.7%) | | 44 (37.6%) | | 9 (47.4%) | | 18 (64.3%) | |
| **Hospitalisation** *N* (%) | 334 (66.0%) | | 117 (89.3%) | | 19 (100.0%) | | 28 (100.0%) | |
| **Length of hospitalisation** (days) (median [Q1-Q4]) | 2 [0 - 7] | | 7 [4 - 12] | | 7 [5 - 8] | | 8 [3.75 - 19.25] | |
| **Infection related readmission** *N* (%) | 28 (5.5%) | | 20 (15.3%) | | 0 (0.0%) | | 3 (10.7%) | |
| **ICU admission** *N* (%) | 9 (1.8%) | | 10 (7.6%) | | 0 (0.0%) | | 4 (14.3%) | |
| **Time to ICU admission** (days) (median [Q1-Q4]) | 13 [2 - 9] | | 1 [0 - 5] | | NA | | 0 [0 - 0] | |
| **Number of immediate ICU admissions** *N* (%) | 2 (8.7%) | | 3 (13.0%) | | 0 (0.0%) | | 4 (17.4%) | |
| **Number of delayed ICU admissions** *N* (%) | 2 (8.7%) | | 6 (26.1%) | | 0 (0.0%) | | 0 (0.0%) | |
| **Number of late ICU admissions** *N* (%) | 5 (21.7%) | | 1 (4.3%) | | 0 (0.0%) | | 0 (0.0%) | |
| **Infection-related 28-day mortality** *N* (%) | 6 (1.2%) | | 17 (13.0%) | | 0 (0.0%) | | 7 (25.0%) | |
| **Hospital mortality** *N* (%) | 8 (1.6%) | | 17 (13.0%) | | 2 (10.5%) | | 8 (28.6%) | |
| **Disease progression** *N* (%) | 32 (6.3%) | | 34 (26.0%) | | 1 (5.3%) | | 12 (42.9%) | |

Number of immediate, delayed or late ICU admissions are expressed as a percentage of the total number of ICU admissions. *ICU*: Intensive Care Unit; *MR-proADM*: Mid-regional proadrenomedullin; *N*: Number; *qSOFA*: quick Sequential Organ Failure Assessment.

**Table S19. Patient subgroups stratified by CRB-65 and MR-proADM**

|  | Patient populations stratified by CRB-65 and MR-proADM | | | | | | | |
| --- | --- | --- | --- | --- | --- | --- | --- | --- |
| **Patient subgroups** | MR-proADM  (nmol/L) | CRB-65  (points) | MR-proADM  (nmol/L) | CRB-65  (points) | MR-proADM  (nmol/L) | CRB-65  (points) | MR-proADM  (nmol/L) | CRB-65  (points) |
|  | <1.77 | <2 | ≥1.77 | <2 | <1.77 | ≥2 | ≥1.77 | ≥2 |
| **Population** *N* (%) | 423 (61.8%) | | 79 (11.5%) | | 102 (14.9%) | | 80 (11.7%) | |
| **Antibiotic administration** *N* (%) | 273 (64.5%) | | 72 (91.1%) | | 87 (85.3%) | | 73 (91.3%) | |
| **Time to antibiotic administration** (mins) (median [Q1-Q4]) | 183 [120 - 330] | | 180 [120 - 300] | | 240 [150 - 355] | | 182 [97.5 - 300] | |
| **Antibiotic administration <180 mins** *N* (%) | 114 (41.8%) | | 30 (41.7%) | | 26 (30.0%) | | 36 (49.3%) | |
| **Hospitalisation** *N* (%) | 262 (61.9%) | | 70 (88.6%) | | 91 (89.2%) | | 75 (93.8%) | |
| **Length of hospitalisation** (days) (median [Q1-Q4]) | 13 [1 - 18] | | 6 [4 - 10.75] | | 6 [2 - 9.5] | | 8 [4 - 16.5] | |
| **Infection related readmission** *N* (%) | 25 (5.9%) | | 15 (19.0%) | | 3 (2.9%) | | 8 (10.0%) | |
| **ICU admission** *N* (%) | 7 (1.7%) | | 5 (6.3%) | | 2 (2.0%) | | 9 (11.3%) | |
| **Time to ICU admission** (days) (median [Q1-Q4]) | 2 [0 - 6] | | 0 [0 - 0] | | NA | | 0 [0 - 2] | |
| **Number of immediate ICU admissions** *N* (%) | 2 (8.7%) | | 3 (13.0%) | | 0 (0.0%) | | 4 (17.4%) | |
| **Number of delayed ICU admissions** *N* (%) | 1 (4.3%) | | 2 (8.7%) | | 1 (4.3%) | | 4 (17.4%) | |
| **Number of late ICU admissions** *N* (%) | 4 (17.4%) | | 0 (0.0%) | | 1 (4.3%) | | 1 (4.3%) | |
| **Infection-related 28-day mortality** *N* (%) | 4 (0.9%) | | 7 (8.9%) | | 2 (2.0%) | | 17 (21.3%) | |
| **Hospital mortality** *N* (%) | 4 (0.9%) | | 7 (8.9%) | | 6 (5.9%) | | 18 (22.5%) | |
| **Disease progression** *N* (%) | 23 (5.4%) | | 18 (22.8%) | | 10 (12.5%) | | 28 (35.0%) | |

Number of immediate, delayed or late ICU admissions are expressed as a percentage of the total number of ICU admissions. *ICU*: Intensive Care Unit; *MR-proADM*: Mid-regional proadrenomedullin; *N*: Number; *CRB-65:* severity sore for community acquired Pneumonia

**Table S20. Patient subgroups based on antibiotic administration using NEWS and MR-proADM**

|  | **Low NEWS, Low MR-proADM subgroup** | | **Low NEWS, High MR-proADM subgroup** | |
| --- | --- | --- | --- | --- |
|  | **Patients receiving antibiotics**  **(*N*=266)** | **Patients not receiving antibiotics**  **(*N*=159)** | **Patients receiving antibiotics**  **(*N*=63)** | **Patients not receiving antibiotics**  **(*N*=11)** |
| Hospitalisation | 225 (84.6%) | 35 (22.0%) | 58 (92.1%) | 5 (45.5%) |
| ICU admission | 6 (2.3%) | 1 (0.6%) | 3 (4.8%) | 3 (27.3%) |
| Hospital readmission | 16 (0.6%) | 9 (5.7%) | 9 (14.3%) | 6 (54.5%) |
| 28-day Mortality | 0 (0.0%) | 0 (0.0%) | 9 (14.3%) | 1 (9.1%) |

Patients with respective NEWS and MR-proADM cut-offs of <5 points and <1.77 nmol/L were designated as “low”, whereas patients with NEWS and MR-proADM cut-offs of ≥5 points and ≥1.77 nmol/L were designated as “high”. *ICU*: Intensive Care Unit; *MR-proADM*: Mid-regional proadrenomedullin; *N*: Number; *NEWS*: National Early Warning Score

**Table S21. Patient subgroups based on antibiotic administration using qSOFA and MR-proADM**

|  | **Low qSOFA, Low MR-proADM subgroup** | | **Low qSOFA, High MR-proADM subgroup** | |
| --- | --- | --- | --- | --- |
|  | **Patients receiving antibiotics**  **(*N*=341)** | **Patients not receiving antibiotics**  **(*N*=165)** | **Patients receiving antibiotics**  **(*N*=117)** | **Patients not receiving antibiotics**  **(*N*=14)** |
| Hospitalisation | 298 (87.4%) | 36 (21.8%) | 110 (94.0%) | 7 (50.0%) |
| ICU admission | 8 (2.3%) | 1 (0.6%) | 6 (5.1%) | 4 (28.6%) |
| Hospital readmission | 19 (5.6%) | 9 (5.4%) | 15 (12.8%) | 5 (35.7%) |
| 28-day Mortality | 6 (1.8%) | 0 (0.0%) | 15 (12.8%) | 2 (14.3%) |

Patients with respective qSOFA and MR-proADM cut-offs of <2 points and <1.77 nmol/L were designated as “low”, whereas patients with qSOFA and MR-proADM cut-offs of ≥2 points and ≥1.77 nmol/L were designated as “high”. *ICU*: Intensive Care Unit; *MR-proADM*: Mid-regional proadrenomedullin; *N*: Number; *qSOFA*: quick Sequential Organ Failure Assessment.

**Table S22. Low NEWS patient subgroups classified according to Lactate and PCT kinetics between ED presentation and 72 hours**

|  | Patient populations stratified by NEWS and either lactate or PCT | | | | | | | |
| --- | --- | --- | --- | --- | --- | --- | --- | --- |
| **Patient subgroups** | Lactate  (mmol/L) | NEWS  (points) | Lactate  (mmol/L) | NEWS  (points) | PCT  (ng/mL) | NEWS  (points) | PCT  (ng/mL) | NEWS  (points) |
|  | <1.90 | <5 | ≥1.90 | <5 | <0.20 | <5 | ≥0.20 | <5 |
| **Population** *N (%)* | 258 (37.7%) | | 101 (14.8%) | | 279 (40.8%) | | 220 (32.2%) | |
| **Infection related 28-day mortality** *N* (%) | 3 (1.2%) | | 7 (6.9%) | | 0 (0.0%) | | 10 (4.5%) | |
| **NEWS: Surviving patients** | | | | | | | | |
| ED admission (points) | 1.83 {1.36) | | 2.05 (1.38) | | 1.54 (1.28) | | 2.11 (1.30) | |
| 72 hours (points) | 1.72 (1.88) | | 1.71 (1.77) | | 1.52 (1.72) | | 1.77 (1.85) | |
| p-value | 0.498 | | 0.202 | | 0.064 | | 0.012 | |
| **NEWS: Non-surviving patients** | | | | | | | | |
| ED admission (points) | 2.67 (1.15) | | 2.57 (0.97) | | NA | | 2.60 (0.97) | |
| 72 hours (points) | 2.67 (3.79) | | 3.14 (1.77) | | NA | | 3.00 (2.31) | |
| p-value | 1.000 | | 0.469 | | NA | | 0.565 | |
| **Biomarker: Surviving patients** | | | | | | | | |
| ED admission | 1.25 (0.33) | | 2.94 (2.26) | | 0.09 (0.05) | | 3.94 (11.05) | |
| 72 hours | 2.22 (3.66) | | 2.38 (3.42) | | 0.17 (0.22) | | 3.67 (11.90) | |
| p-value | <0.001 | | 0.284 | | 0.010 | | 0.268 | |
| **Biomarker: Non-surviving patients** | | | | | | | | |
| ED admission | 1.33 (0.29) | | 2.93 (1.78) | | NA | | 9.92 (15.65) | |
| 72 hours | 1.26 (0.12) | | 1.97 (0.23) | | NA | | 6.64 (8.71) | |
| p-value | 0.691 | | 0.183 | | NA | | 0.553 | |

*ED*: Emergency Department; *N*: Number; *NEWS*: National Early Warning Score; *PCT*: Procalcitonin;

**Table S23. qSOFA and MR-proADM values upon presentation and 72 hours within stratified subgroups**

|  | | | Patient populations stratified by qSOFA and MR-proADM | | | | | | | |
| --- | --- | --- | --- | --- | --- | --- | --- | --- | --- | --- |
| **Patient subgroups** | | | MR-proADM  (nmol/L) | qSOFA  (points) | MR-proADM  (nmol/L) | qSOFA  (points) | MR-proADM  (nmol/L) | qSOFA  (points) | MR-proADM  (nmol/L) | qSOFA  (points) |
|  |  |  | <1.77 | <2 | ≥1.77 | <2 | <1.77 | ≥2 | ≥1.77 | ≥2 |
| **Population**  *N* (%) | | | 506 (74.0%) | | 131 (19.2%) | | 19 (2.8%) | | 28 (4.1%) | |
| **Infection related 28-day mortality**  *N* (%) | | | 6 (1.2%) | | 17 (13.0%) | | 0 (0.0%) | | 7 (25.0%) | |
| **Disease progression: qSOFA** | **Total cohort** | Admission (points) | 0.44 (0.50) | | 0.61 (0.49) | | 2.00 (0.00) | | 2.20 (0.42) | |
|  |  | 72 hours (points) | 0.38 (0.61) | | 0.51 (0.69) | | 0.87 (0.63) | | 0.87 (0.63) | |
|  |  | p-value | 0.749 | | 0.115 | | <0.001 | | <0.001 | |
|  | **Surviving patients** | Admission (points) | 0.41 (0.50) | | 0.53 (0.50) | | 2.24 (0.44) | | 2.24 (0.44) | |
|  |  | 72 hours (points) | 0.38 (0.61) | | 0.45 (0.65) | | 0.95 (0.62) | | 0.95 (0.62) | |
|  |  | p-value | 0.867 | | 0.184 | | <0.001 | | <0.001 | |
|  | **Non-surviving patients** | Admission (points) | 0.60 (0.55) | | 0.87 (0.35) | | NA | | 2.14 (0.38) | |
|  |  | 72 hours (points) | 0.80 (0.84) | | 0.60 (0.74) | | NA | | 0.50 (0.58) | |
|  |  | p-value | 0.704 | | 0.165 | | NA | | 0.014 | |
| **Disease progression: MR-proADM** | **Total cohort** | Admission (nmol/L) | 1.17 (0.38) | | 3.11 (1.28) | | 1.37 (0.32) | | 3.63 (1.82) | |
|  |  | 72 hours (nmol/L) | 1.03 (0.48) | | 2.35 (1.58) | | 1.24 (0.47) | | 2.23 (1.64) | |
|  |  | p-value | 0.010 | | 0.001 | | 0.348 | | 0.002 | |
|  | **Surviving patients** | Admission (nmol/L) | 1.68 (0.37) | | 3.09 (1.24) | | 1.37 (0.32) | | 3.82 (1.79) | |
|  |  | 72 hours (nmol/L) | 1.01 (0.48) | | 2.04 (1.07) | | 1.24 (0.47) | | 2.29 (1.85) | |
|  |  | p-value | 0.010 | | <0.001 | | 0.348 | | 0.002 | |
|  | **Non-surviving patients** | Admission (nmol/L) | 1.30 (0.29) | | 2.98 (1.16) | | NA | | 2.20 (0.46) | |
|  |  | 72 hours (nmol/L) | 0.94 (0.48) | | 3.17 (2.53) | | NA | | 2.04 (0.60) | |
|  |  | p-value | 0.256 | | 0.617 | | NA | | 0.467 | |

All NEWS and MR-proADM values are expressed as mean (standard deviation). *MR-proADM*: Mid-regional proadrenomedullin; *N*: Number; *qSOFA*: quick Sequential Organ Failure Assessment.

**Supplementary Figures**


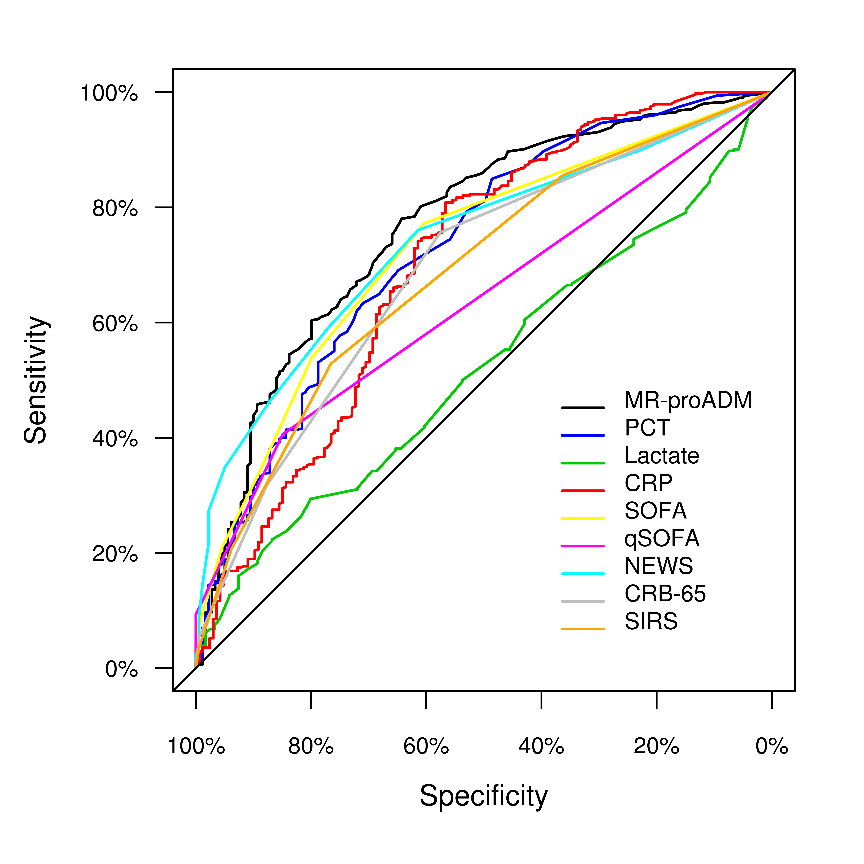
**Figure S1. AUROC analysis for antibiotic requirement during treatment within the ED**

|  | **Biomarkers and clinical scores** | **AUC** | **Cut-off** | **Sensitivity** | **Specificity** | **PPV** | **NPV** | **LR+** | **LR-** | **OR** |
| --- | --- | --- | --- | --- | --- | --- | --- | --- | --- | --- |
| ED admission | MR-proADM | 0.77 [0.73-0.81] | 0.80 | 0.78 [0.74-0.81] | 0.64 [0.57-0.71] | 0.86 [0.83-0.89] | 0.51 [0.44-0.57] | 2.18 [1.78-2.67] | 0.34 [0.28-0.42] | 6.4 [4.4 - 9.2] |
|  | PCT | 0.73 [0.69-0.77] | 0.18 | 0.63 [0.59-0.67] | 0.71 [0.64-0.77] | 0.86 [0.82-0.89] | 0.41 [0.35-0.46] | 2.18 [1.72-2.77] | 0.52 [0.45-0.60] | 4.3 [2.9 - 6.2] |
|  | Lactate | 0.52 [0.47-0.58] | 2.06 | 0.29 [0.25-0.34] | 0.80 [0.72-0.86] | 0.83 [0.77-0.89] | 0.25 [0.21-0.30] | 1.48 [1.00-2.18] | 0.88 [0.79-0.98] | 1.6 [1.0 - 2.7] |
|  | CRP | 0.70 [0.65-0.75] | 4.07 | 0.81 [0.77-0.84] | 0.57 [0.49-0.64] | 0.84 [0.81-0.87] | 0.51 [0.43-0.58] | 1.86 [1.56-2.23] | 0.34 [0.27-0.42] | 5.5 [3.8 - 8.1] |
|  | SOFA | 0.73 [0.69-0.77] | 1 | 0.77 [0.73-0.81] | 0.60 [0.53-0.67] | 0.85 [0.81-0.88] | 0.48 [0.42-0.55] | 1.95 [1.62-2.35] | 0.38 [0.31-0.46] | 5.2 [3.6 - 7.4] |
|  | qSOFA | 0.64 [0.60-0.67] | 1 | 0.41 [0.36-0.45] | 0.85 [0.79-0.89] | 0.88 [0.84-0.92] | 0.34 [0.29-0.38] | 2.69 [1.87-3.87] | 0.70 [0.64-0.77] | 3.8 [2.5 - 6.0] |
|  | NEWS | 0.74 [0.70-0.78] | 2 | 0.76 [0.72-0.80] | 0.61 [0.54-0.68] | 0.85 [0.81-0.88] | 0.48 [0.41-0.54] | 1.97 [1.63-2.39] | 0.39 [0.32-0.47] | 5.1 [3.5 - 7.3] |
|  | CRB-65 | 0.69 [0.65-0.73] | 1 | 0.76 [0.72-0.79] | 0.58 [0.50-0.65] | 0.83 [0.80-0.87] | 0.46 [0.39-0.52] | 1.78 [1.49-2.13] | 0.42 [0.35-0.52] | 4.2 [2.9 - 6.0] |
|  | SIRS | 0.69 [0.64-0.73] | 2 | 0.53 [0.49-0.57] | 0.77 [0.70-0.82] | 0.86 [0.82-0.90] | 0.37 [0.32-0.42] | 2.25 [1.71-2.97] | 0.62 [0.54-0.70] | 3.7 [2.5 - 5.4] |

*CRB-65*: Severity score for community-acquired pneumonia; *CRP*: C-reactive protein; *MR-proADM*: Mid-regional proadrenomedullin; *NEWS*: National Early Warning Score; *PCT*: Procalcitonin; *qSOFA*: quick Sequential Organ Failure Assessment; *SOFA*: Sequential Organ Failure Assessment.

**Figure S2. AUROC analysis for hospitalisation requirement upon ED presentation**


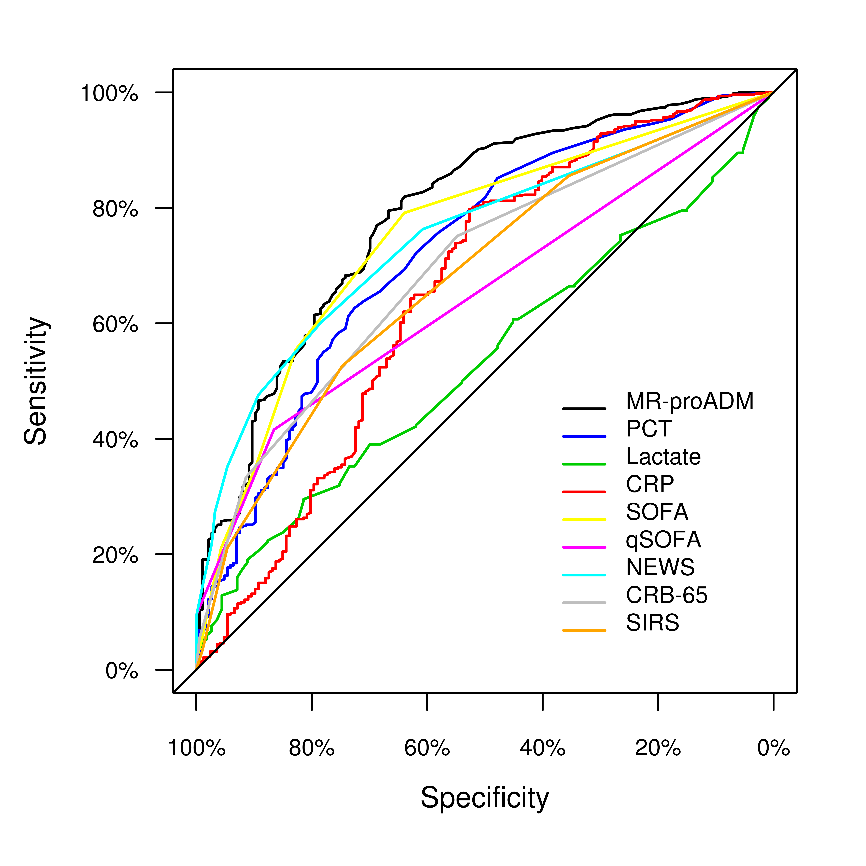


|  | **Biomarkers and clinical scores** | **AUC** | **Cut-off** | **Sensitivity** | **Specificity** | **PPV** | **NPV** | **LR+** | **LR-** | **OR** |
| --- | --- | --- | --- | --- | --- | --- | --- | --- | --- | --- |
| ED admission | MR-proADM | 0.79 [0.75-0.83] | 0.8 | 0.80 [0.76-0.83] | 0.67 [0.60-0.73] | 0.86 [0.83-0.89] | 0.55 [0.48-0.61] | 2.39 [1.94-2.94] | 0.31 [0.25-0.38] | 7.8 [5.3 - 11.3] |
|  | PCT | 0.73 [0.68-0.77] | 0.19 | 0.63 [0.58-0.67] | 0.73 [0.66-0.78] | 0.86 [0.82-0.89] | 0.42 [0.37-0.48] | 2.28 [1.79-2.91] | 0.51 [0.45-0.59] | 4.4 [3.1 - 6.4] |
|  | Lactate | 0.53 [0.48-0.59] | 2.06 | 0.30 [0.25-0.34] | 0.81 [0.73-0.88] | 0.86 [0.79-0.90] | 0.24 [0.20-0.28] | 1.59 [1.05-2.40] | 0.87 [0.78-0.96] | 1.8 [1.1 - 3.1] |
|  | CRP | 0.66 [0.61-0.71] | 3.97 | 0.80 [0.76-0.83] | 0.53 [0.45-0.60] | 0.83 [0.79-0.86] | 0.48 [0.40-0.55] | 1.69 [1.43-1.99] | 0.38 [0.31-0.48] | 4.4 [3.0 - 6.4] |
|  | SOFA | 0.75 [0.71-0.79] | 1 | 0.79 [0.75-0.82] | 0.64 [0.57-0.71] | 0.85 [0.82-0.88] | 0.53 [0.47-0.60] | 2.20 [1.80-2.67] | 0.33 [0.27-0.40] | 6.7 [4.7 - 9.7] |
|  | qSOFA | 0.65 [0.62-0.68] | 1 | 0.42 [0.37-0.46] | 0.87 [0.81-0.91] | 0.89 [0.85-0.93] | 0.36 [0.31-0.40] | 3.09 [2.12-4.52] | 0.68 [0.61-0.74] | 4.6 [2.9 - 7.2] |
|  | NEWS | 0.75 [0.71-0.79] | 3 | 0.60 [0.56-0.64] | 0.78 [0.72-0.84] | 0.88 [0.84-0.91] | 0.42 [0.37-0.48] | 2.79 [2.10-3.71] | 0.51 [0.45-0.58] | 5.5 [3.7 - 8.1] |
|  | CRB-65 | 0.69 [0.66-0.73] | 1 | 0.75 [0.71-0.79] | 0.55 [0.48-0.62] | 0.82 [0.78-0.85] | 0.45 [0.39-0.52] | 1.66 [1.41-1.96] | 0.45 [0.37-0.55] | 3.7 [2.6 - 5.2] |
|  | SIRS | 0.68 [0.64-0.72] | 2 | 0.53 [0.48-0.57] | 0.75 [0.68-0.80] | 0.85 [0.80-0.88] | 0.37 [0.32-0.42] | 2.08 [1.60-2.70] | 0.63 [0.56-0.72] | 3.3 [2.3 - 4.8] |

*CRB-65*: Severity score for community-acquired pneumonia; *CRP*: C-reactive protein; *MR-proADM*: Mid-regional proadrenomedullin; *NEWS*: National Early Warning Score; *PCT*: Procalcitonin; *qSOFA*: quick Sequential Organ Failure Assessment; *SOFA*: Sequential Organ Failure Assessment.

**Figure S3. AUROC analysis for ICU admission within 28 days of initial ED presentation**


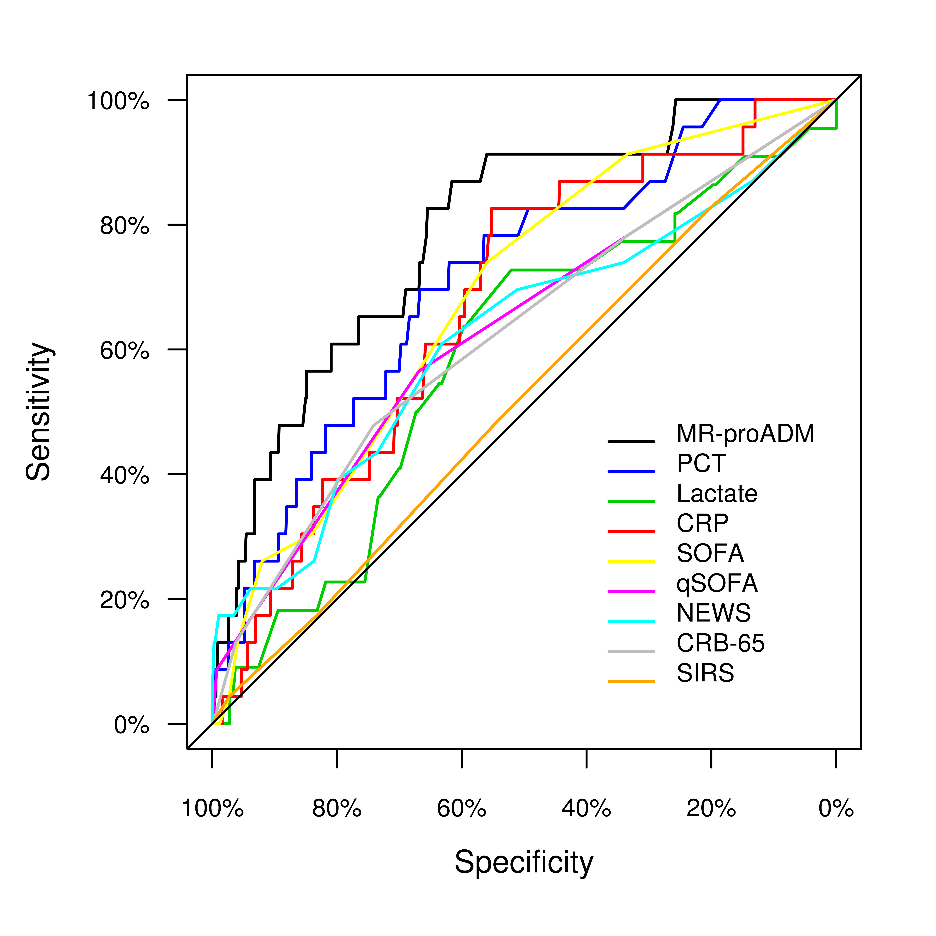


|  | **Biomarkers and clinical scores** | **AUC** | **Cut-off** | **Sensitivity** | **Specificity** | **PPV** | **NPV** | **LR+** | **LR-** | **OR** |
| --- | --- | --- | --- | --- | --- | --- | --- | --- | --- | --- |
| ED admission | MR-proADM | 0.79 [0.70-0.88] | 1.26 | 0.87 [0.68-0.95] | 0.62 [0.58-0.65] | 0.07 [0.05-0.11] | 0.99 [0.98-1.00] | 2.26 [1.88-2.72] | 0.21 [0.07-0.61] | 10.7 [3.1 - 36.3] |
|  | PCT | 0.71 [0.61-0.82] | 0.47 | 0.70 [0.49-0.84] | 0.67 [0.63-0.70] | 0.07 [0.04-0.11] | 0.98 [0.97-0.99] | 2.09 [1.56-2.80] | 0.46 [0.25-0.85] | 4.6 [1.9 - 11.3] |
|  | Lactate | 0.59 [0.47-0.71] | 1.6 | 0.73 [0.52-0.87] | 0.52 [0.48-0.56] | 0.06 [0.04-0.10] | 0.98 [0.95-0.99] | 1.52 [1.16-1.99] | 0.52 [0.26-1.04] | 2.9 [1.1 - 7.5] |
|  | CRP | 0.67 [0.57-0.78] | 12.2 | 0.83 [0.63-0.93] | 0.55 [0.51-0.59] | 0.06 [0.04-0.10] | 0.99 [0.97-1.00] | 1.84 [1.50-2.27] | 0.31 [0.13-0.77] | 5.9 [2.0 - 17.4] |
|  | SOFA | 0.68 [0.58-0.78] | 2 | 0.74 [0.54-0.87] | 0.56 [0.52-0.60] | 0.06 [0.03-0.09] | 0.98 [0.97-0.99] | 1.68 [1.30-2.17] | 0.47 [0.23-0.93] | 3.6 [1.4 - 9.2] |
|  | qSOFA | 0.63 [0.52-0.74] | 1 | 0.57 [0.37-0.74] | 0.67 [0.63-0.70] | 0.06 [0.03-0.09] | 0.98 [0.96-0.99] | 1.71 [1.17-2.48] | 0.65 [0.41-1.04] | 2.6 [1.1 - 6.1] |
|  | NEWS | 0.62 [0.49-0.75] | 4 | 0.61 [0.41-0.78] | 0.63 [0.59-0.67] | 0.05 [0.03-0.09] | 0.98 [0.96-0.99] | 1.66 [1.18-2.33] | 0.62 [0.37-1.03] | 2.7 [1.1 - 6.3] |
|  | CRB-65 | 0.62 [0.53-0.75] | 2 | 0.48 [0.29-0.67] | 0.74 [0.71-0.77] | 0.06 [0.03-0.10] | 0.98 [0.96-0.99] | 1.85 [1.18-2.89] | 0.70 [0.47-1.04] | 2.6 [1.1 - 6.1] |
|  | SIRS | 0.52 [0.40-0.64] | 1 | 0.83 [0.63-0.93] | 0.20 [0.17-0.24] | 0.03 [0.02-0.05] | 0.97 [0.93-0.99] | 1.04 [0.86-1.25] | 0.86 [0.35-2.12] | 1.2 [0.4 - 3.6] |

*CRB-65*: Severity score for community-acquired pneumonia; *CRP*: C-reactive protein; *MR-proADM*: Mid-regional proadrenomedullin; *NEWS*: National Early Warning Score; *PCT*: Procalcitonin; *qSOFA*: quick Sequential Organ Failure Assessment; *SOFA*: Sequential Organ Failure Assessment.

**Figure S4. AUROC analysis for infection-related 28-day mortality upon ED presentation and 72 hours**


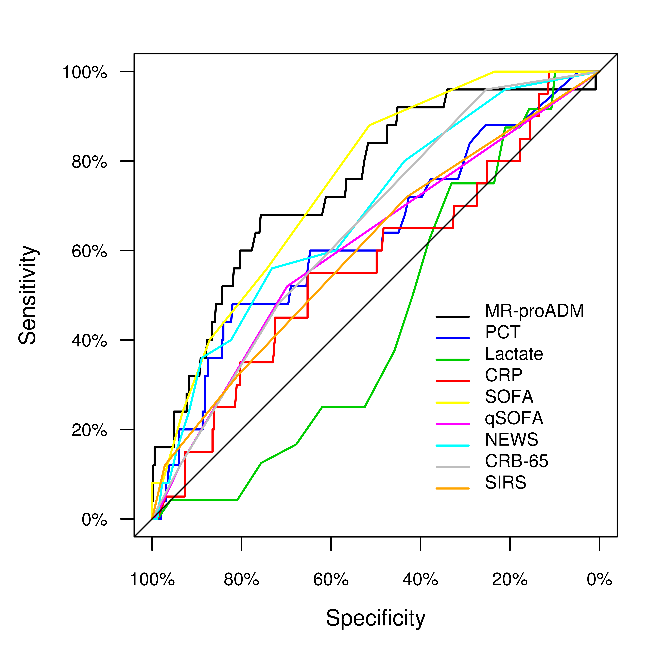

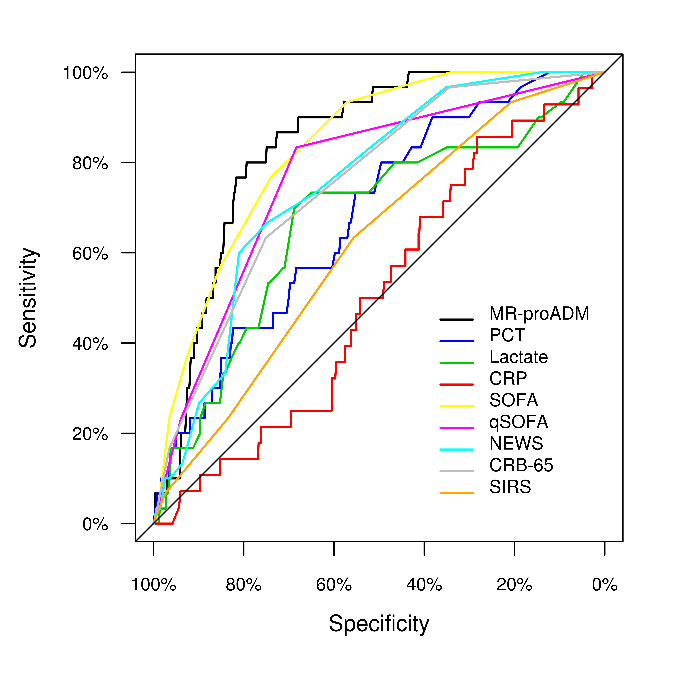


**B**

**A**

|  | **Biomarkers and clinical scores** | **AUC** | **Cut-off** | **Sensitivity** | **Specificity** | **PPV** | **NPV** | **LR+** | **LR-** | **OR** |
| --- | --- | --- | --- | --- | --- | --- | --- | --- | --- | --- |
| ED admission | MR-proADM | 0.84 [0.79-0.89] | 1.77 | 0.80 [0.63-0.90] | 0.79 [0.76-0.82] | 0.15 [0.10-0.21] | 0.99 [0.98-0.99] | 3.88 [3.07-4.90] | 0.25 [0.12-0.52] | 15.4 [6.2 - 38.4] |
|  | PCT | 0.68 [0.59-0.77] | 0.2 | 0.80 [0.63-0.90] | 0.50 [0.46-0.53] | 0.07 [0.05-0.10] | 0.98 [0.96-0.99] | 1.59 [1.31-1.93] | 0.40 [0.20-0.83] | 3.9 [1.6 - 9.7] |
|  | Lactate | 0.67 [0.57-0.78] | 1.9 | 0.70 [0.52-0.83] | 0.69 [0.65-0.73] | 0.12 [0.08-0.17] | 0.97 [0.95-0.99] | 2.24 [1.72-2.93] | 0.44 [0.25-0.76] | 5.1 [2.3 - 11.5] |
|  | CRP | 0.51 [0.41-0.60] | 7 | 0.75 [0.57-0.87] | 0.39 [0.36-0.43] | 0.05 [0.04-0.08] | 0.97 [0.94-0.99] | 1.24 [0.99-1.55] | 0.63 [0.33-1.21] | 2.0 [0.8 - 4.7] |
|  | SOFA | 0.83 [0.77-0.89] | 3 | 0.77 [0.59-0.88] | 0.74 [0.71-0.77] | 0.12 [0.08-0.17] | 0.99 [0.97-0.99] | 2.95 [2.33-3.73] | 0.32 [0.16-0.60] | 9.4 [3.9 - 22.2] |
|  | qSOFA | 0.77 [0.69-0.85] | 1 | 0.83 [0.66-0.93] | 0.68 [0.65-0.72] | 0.11 [0.07-0.15] | 0.99 [0.97-1.00] | 2.63 [2.16-3.20] | 0.24 [0.11-0.54] | 10.8 [4.1 - 28.6] |
|  | NEWS | 0.75 [0.68-0.83] | 5 | 0.67 [0.49-0.81] | 0.75 [0.71-0.78] | 0.11 [0.07-0.16] | 0.98 [0.96-0.99] | 2.64 [1.99-3.52] | 0.45 [0.27-0.74] | 5.9 [2.7 - 12.9] |
|  | CRB-65 | 0.75 [0.68-0.83] | 2 | 0.63 [0.46-0.78] | 0.75 [0.72-0.78] | 0.10 [0.07-0.16] | 0.98 [0.96-0.99] | 2.54 [1.88-3.44] | 0.49 [0.30-0.78] | 5.2 [2.4 - 11.2] |
|  | SIRS | 0.62 [0.53-0.71] | 2 | 0.63 [0.46-0.78] | 0.56 [0.52-0.59] | 0.06 [0.04-0.09] | 0.97 [0.95-0.98] | 1.43 [1.07-1.90] | 0.66 [0.41-1.06] | 2.2 [1.0 - 4.6] |
| 72 hours | MR-proADM | 0.75 [0.65-0.85] | 1.57 | 0.68 [0.48-0.83] | 0.76 [0.71-0.80] | 0.17 [0.11-0.25] | 0.97 [0.94-0.99] | 2.80 [2.02-3.88] | 0.42 [0.24-0.75] | 6.6 [2.8 - 15.9] |
|  | PCT | 0.63 [0.51-0.75] | 1.78 | 0.48 [0.30-0.67] | 0.82 [0.78-0.86] | 0.16 [0.10-0.26] | 0.96 [0.93-0.97] | 2.67 [1.68-4.26] | 0.63 [0.43-0.93] | 4.2 [1.8 - 9.7] |
|  | Lactate | 0.56 [0.46-0.66] | 1.7 | 0.75 [0.55-0.88] | 0.48 [0.41-0.54] | 0.12 [0.08-0.19] | 0.95 [0.90-0.98] | 1.43 [1.10-1.85] | 0.53 [0.26-1.07] | 2.7 [1.0 - 7.1] |
|  | CRP | 0.57 [0.43-0.71] | 16.4 | 0.30 [0.15-0.52] | 0.73 [0.67-0.78] | 0.08 [0.04-0.16] | 0.93 [0.89-0.96] | 1.09 [0.54-2.20] | 0.96 [0.72-1.30] | 1.1 [0.4 - 3.1] |
|  | SOFA | 0.75 [0.67-0.84] | 2 | 0.88 [0.70-0.96] | 0.51 [0.46-0.57] | 0.11 [0.08-0.17] | 0.98 [0.95-0.99] | 1.81 [1.51-2.17] | 0.23 [0.08-0.68] | 7.8 [2.3 - 26.4] |
|  | qSOFA | 0.61 [0.51-0.72] | 1 | 0.52 [0.33-0.70] | 0.70 [0.65-0.74] | 0.11 [0.06-0.18] | 0.95 [0.92-0.97] | 1.72 [1.14-2.59] | 0.69 [0.45-1.04] | 2.5 [1.1 - 5.7] |
|  | NEWS | 0.68 [0.58-0.79] | 4 | 0.56 [0.37-0.73] | 0.73 [0.68-0.78] | 0.13 [0.08-0.21] | 0.96 [0.93-0.98] | 2.09 [1.42-3.08] | 0.60 [0.38-0.94] | 3.5 [1.5 - 7.9] |
|  | CRB-65 | 0.65 [0.56-0.74] | 1 | 0.96 [0.80-0.99] | 0.25 [0.21-0.30] | 0.08 [0.06-0.12] | 0.99 [0.94-1.00] | 1.29 [1.16-1.42] | 0.16 [0.02-1.09] | 2.2 [1.0 - 5.0] |
|  | SIRS | 0.60 [0.49-0.72] | 1 | 0.72 [0.52-0.86] | 0.43 [0.38-0.48] | 0.08 [0.05-0.13] | 0.96 [0.91-0.98] | 1.26 [0.97-1.64] | 0.65 [0.34-1.23] | 1.9 [0.8 - 4.8] |

*CRB-65*: Severity score for community-acquired pneumonia; *CRP*: C-reactive protein; *MR-proADM*: Mid-regional proadrenomedullin; *NEWS*: National Early Warning Score; *PCT*: Procalcitonin; *qSOFA*: quick Sequential Organ Failure Assessment; *SOFA*: Sequential Organ Failure Assessment.
